# Microglia from patients with multiple sclerosis display a cell-autonomous immune activation state

**DOI:** 10.1186/s12974-025-03575-4

**Published:** 2025-10-31

**Authors:** Tanja Hyvärinen, Johanna Tilvis, Luca Giudice, Iisa Tujula, Marjo Nylund, Sohvi Ohtonen, Flavia Scoyni, Henna Jäntti, Lassi Virtanen, Sara Pihlava, Roosa Kattelus, Heli Skottman, Susanna Narkilahti, Laura Airas, Tarja Malm, Sanna Hagman

**Affiliations:** 1 Neuroimmunology research group, Faculty of Medicine and Health Technology, Tampere University, Arvo Ylpön katu 34, Tampere, 33520 Finland; 2 Neuroinflammation research group, Faculty of Health Sciences, A.I. Virtanen Institute for Molecular Sciences, University of Eastern Finland, Kuopio, Finland; 3 Clinical neurosciences, University of Turku, Turku, Finland; 4 Neurocenter, Turku University Hospital, Turku, Finland; 5 Turku PET Centre, University of Turku, Turku University Hospital and Åbo Akademi University, Turku, Finland; 6 InFLAMES Research Flagship, University of Turku, Turku, Finland; 7 Systems Biology of Gene Regulatory Elements, Max Delbrück Center for Molecular Medicine in the Helmholtz Association (MDC), Berlin Institute for Medical Systems Biology (BIMSB), Berlin, Germany; 8 Eye Regeneration group, Faculty of Medicine and Health Technology, Tampere University, Tampere, Finland; 9 NeuroGroup, Faculty of Medicine and Health Technology, Tampere University, Tampere, Finland

**Keywords:** Human induced pluripotent stem cells, Microglia, Multiple sclerosis, Neuroinflammation

## Abstract

Aberrant and sustained activation of microglia is implicated in the progression and severity of multiple sclerosis (MS). However, whether intrinsic alterations in microglial function impact the pathogenesis of this disease remains unclear. We conducted transcriptomic and functional analyses of microglia-like cells (iMGLs) differentiated from induced pluripotent stem cells (iPSCs) from patients with MS (pwMS) to answer this question. The pwMS showed increased innate immune cell activity via 18-kDa translocator protein positron emission tomography imaging. After confirming that the differentiated iMGLs transcriptional profile is determined by the microglial cell type, comparative studies were performed to identify the transcriptional and functional differences between iMGLs from pwMS and healthy controls. Importantly, MS iMGLs presented cell-autonomous differences in their regulation of inflammation, both in the basal state and following inflammatory lipopolysaccharide challenge. Through transcriptomic profiling, we showed that MS iMGLs display increased expression of genes upregulated in MS pathology. Furthermore, upregulated genes in MS iMGLs were associated with immune receptor activation, antigen presentation, and the complement system. MS iMGLs demonstrated transcriptional similarities to lesion-specific microglia in MS, marked by upregulation of immune-related genes and pathways, including those involved in antigen presentation. Finally, functional analyses indicated that the transcriptional changes in MS iMGLs corresponded with modulation of cytokine secretion and increased phagocytosis. Together, our results provide evidence of putative cell-autonomous microglial activation in pwMS and identify transcriptomic and functional changes that recapitulate the phenotypes observed in vivo in microglia from pwMS. These findings indicate that MS disease-specific iPSCs are valuable tools for studying disease-specific microglial activation in vitro and highlight microglia as potential therapeutic targets in MS.

**Supplementary Information:**

The online version contains supplementary material available at 10.1186/s12974-025-03575-4.

## Background

Multiple sclerosis (MS) is a chronic autoimmune disease of the central nervous system (CNS) characterized by focal demyelinated lesions, inflammation and neurodegeneration [1]. Both environmental and lifestyle factors along with genetic predisposition influence the risk of developing MS [2]. Recent genome-wide association studies (GWASs) have identified more than 200 susceptibility variants for MS [3]. Interestingly, these identified susceptibility variants are enriched not only in peripheral immune cells but also in microglia, which are the brain-resident immune cells, suggesting a role in disease onset [3]. Furthermore, newly discovered severity variants link for the first time, faster MS progression and increased cortical pathology to CNS cell functions. This finding underscores the importance of CNS-specific pathological mechanisms in modulating the severity of MS [4].

From the onset of the disease, underlying pathological processes within the CNS contribute to the development of disability [5–7]. Disease progression is associated with the presence of chronic active lesions characterized by a hypocellular centre surrounded by activated microglia at the lesion edge [1, 8, 9]. Many of these lesions can also be identified by the presence of iron-laden microglia and macrophages using brain magnetic resonance imaging (MRI) which can predict more severe tissue damage and worse clinical outcomes [10, 11]. Similarly, positron emission tomography (PET) imaging of the mitochondrial 18-kDa translocator protein (TSPO) ligand has been utilized to study innate immune cell involvement in vivo [12]. PET studies have shown that innate immune cell activity precedes signs of neurodegeneration [13] and is associated with faster disease progression [14]. Thus, this evidence suggests that microglia play a critical role in the immunopathological processes of MS. However, the underlying mechanisms and their contributions to the chronic stages remain uncertain.

Microglia are highly dynamic, phagocytic cells that constantly survey and maintain tissue homeostasis in the CNS. Under pathological conditions, they react and transform into different functional states according to context-dependent signals, including the local immune environment and the functions of other cells [15–17]. However, over time, the initial resolving immune response can escalate into a vicious cycle of chronic neuroinflammation with a potential loss of homeostatic functions and increased production of neurotoxic factors, including proinflammatory cytokines, chemokines, nitric oxide and reactive oxygen species [18]. Aberrant and sustained inflammatory responses of microglia have been documented in experimental animal models of MS and histopathological postmortem brain tissues from patients with MS (pwMS) [19–23]. Studies have reported the loss of homeostatic microglial markers and the presence of activated microglia in various MS lesion types and normal-appearing white matter (NAWM) from autopsy tissues of pwMS [24, 25]. This microglial activation becomes more pronounced with increasing disease duration [24]. Notably, clusters of activated microglia, known as microglial nodules, are present in the NAWM before lesion formation [25, 26].

Single-cell (sc) and single-nucleus (sn) RNA-sequencing (RNA-seq) studies of postmortem brain tissue from pwMS have provided valuable insights into disease-associated microglial states [20, 21, 25, 27–29]. These studies identified gene signatures involved in immune regulation, lipid metabolism, iron homeostasis, phagocytosis, the complement system and antigen presentation [20, 21, 25, 27]. A recent seminal study described two distinct phenotypes of microglia inflamed in MS (MIMS), one referred to as MIMS-foamy, which is involved in the phagocytosis and clearance of myelin, and the other, MIMS-iron, which is characterized by the enrichment of iron- and complement-related genes [20]. Despite these advancements, it remains unknown whether MS microglia exhibit cell-autonomous alterations that contribute to disease susceptibility and disease processes, such as lesion formation or progression. Understanding microglial dysfunction in MS is crucial for developing targeted therapies.

Stem cell technology represents a complementary research tool to traditional disease models. Human induced pluripotent stem cells (iPSCs) can be generated from pwMS, providing unique opportunities to study this disease [30]. To date, studies of MS iPSCs have revealed several key findings: the senescence of neural progenitors [31], deficits in oligodendrocyte progenitor cell migration [32] and differentiation in an inflammatory milieu [33], increased inflammatory activation of astrocytes [34], altered metabolic function of astrocytes [35], and disrupted barrier function of microvascular endothelial cells [36]. In recent years, pioneering studies have reported methods to produce microglia-like cells (iMGLs) from iPSCs, addressing the previously unmet need for in vitro models of microglial phenotypes in neurological disorders, including MS [37–40]. In this study, we aimed to determine whether MS patient-derived iMGLs exhibit cell-intrinsic alterations in their homeostatic and immune activation states. We investigated these properties by generating iPSC lines from six pwMS showing innate immune cell activation using TSPO-PET imaging, differentiated them into iMGLs and compared them to healthy controls (HCs) by performing RNA-seq and functional assays, including secretome and phagocytosis analyses. We found that MS patient-derived iMGLs displayed a distinct inflammatory phenotype at the transcriptome level, which translated into functional changes. These changes included modulation of cytokine and chemokine secretion and increased phagocytic activity. These findings suggest that patient-specific iMGLs represent an exceptional tool for modelling microglial dysfunction in MS and may inform therapeutic strategies that focus on targeting microglia.

## Materials and methods

### Subjects and procedures

Six pwMS were recruited during 2020 from the Turku MS PET cohort (Table 1). Two patients had relapsing-remitting MS (RRMS), and four had secondary progressive MS (SPMS). The Turku MS PET cohort consists of over 150 patients with different stages of MS who have participated in different MS PET studies at the Turku PET centre and have been examined using PET, MRI and clinical evaluation between 2009 and 2024. All participants were interviewed and clinically examined by a neurologist. The Expanded Disability Status Scale (EDSS) score [41] was assessed for patients. The inclusion criteria were a confirmed MS diagnosis according to the 2010 McDonald criteria [42], a previous PET scan using the [^11^C]PK11195 radioligand and female sex. Patients provided informed consent, after which a blood sample was obtained for iPSC reprogramming. For comparison, six similarly imaged age- and sex-matched healthy controls were included for MRI and PET imaging analyses (Table 1). The study protocol was approved by the Ethics Committee of the Hospital District of Southwest Finland (Dnro: 48/1801/2019) and the study was conducted in accordance with the principles of the Declaration of Helsinki.

### MRI and PET data acquisition and processing

Imaging was performed at the Turku University Hospital Neurocenter and Turku PET Centre. Conventional brain MRI using a 3T-MRI (Philips Ingenuity, Best, The Netherlands) was obtained with the following sequences: 3DT1, T2 and fluid-attenuated inversion recovery (FLAIR). Preliminary region of interest (ROI) masks for T1 lesions and FLAIR-based T2 lesions were created via the lesion segmentation toolbox (LST) [43] in SPM12 (Wellcome Trust Center for Neuroimaging, London, UK) and manually corrected after visual inspection to correspond to the T1 and T2 lesions. The whole brain, white matter (WM), grey matter (GM) and thalamic volumes were segmented after lesion filling using FreeSurfer (https://surfer.nmr.mgh.harvard.edu/). NAWM masks were obtained by subtracting the T2-lesion mask from the respective WM mask. Volumes of each ROI were evaluated as parenchymal fractions (PFs). Sixty-minute dynamic brain PET scans were performed using a brain-dedicated High-Resolution Research Tomograph scanner (HRRT, Siemens/Control Technology Incorporated, Knoxville, TN). The radiochemical synthesis of [^11^C]PK11195, image reconstruction and post-processing were performed as previously described [44]. Each subject’s PET image was co-registered with the respective T1 image using statistical parametric mapping (SPM8, Wellcome Trust Center for Neuroimaging). Specific binding of the radioligand was quantified as the distribution volume ratio (DVR). Time–activity curves corresponding to a reference region with no specific binding were obtained using Super-PK-software in MATLAB (MathWorks Inc.) [45], and Logan’s reference tissue model [46] was applied for the DVR estimation.

### Generation and maintenance of iPSCs

Seven HC iPSC lines were utilized in this study. The control iPSC lines UTA.04511.WT [47], UTA.04602.WT [47], UTA.10,902.EURCCs [48] and UTA.11,311.EURCCs [49] were previously generated and characterized at Tampere University with the approval of the Ethics Committee of Wellbeing Services County of Pirkanmaa (R08070, R12123). The control iPSC lines Hel54.1, Hel55.5 and Hel96.6 [50] were obtained from the Finnish Institute for Health and Welfare. Six MS iPSC lines were generated as previously described [51]. One of the MS iPSC lines can also be found at the Human Pluripotent Stem Cell Registry (https://hpscreg.eu) with the hPSCreg name TAUi008-A. For this study, supportive ethical statements for producing MS patient-derived iPSC lines (48/1801/2019) and for culturing and differentiating iPSCs for neuronal research (R20159) were obtained from the Ethics Committee of the Hospital District of Southwest Finland and the Wellbeing Services County of Pirkanmaa, respectively. Informed consent was obtained from all the subjects. The details of the iPSC lines are presented in Supplementary Table 1. The control iPSCs were previously derived from peripheral blood mononuclear cells (PBMCs) or fibroblasts using Sendai virus [47–50] or retrovirus reprogramming [47]. The previously uncharacterized MS iPSCs were produced from PBMCs using the CytoTune™-iPS 2.0 Sendai Reprogramming Kit (Thermo Fisher Scientific). The iPSC lines were derived on a feeder layer of mitomycin C-inactivated human foreskin fibroblasts (CRL-2429™, ATCC). All iPSC lines were subsequently maintained as previously described [48] in feeder-free cultures on 0.6 µg/cm^2^ recombinant human laminin-521 (LN521, Biolamina)-coated cell culture plates in Essential 8™ Flex medium (E8 flex, Thermo Fisher Scientific). IPSCs were passaged enzymatically with TrypLE™ Select Enzyme and Defined Trypsin Inhibitor (both from Thermo Fisher Scientific) in the presence of 10 µM ROCK inhibitor (ROCKi, Y-27632, StemCell Technologies) twice a week.

### Characterization of MS iPSC lines

MS iPSC lines were characterized as previously described [51]. Briefly, the expression of pluripotency markers (Oct3/4, Sox2, Nanog, and SSEA4) was analysed via immunofluorescence staining and flow cytometry. The trilineage differentiation capacity of iPSCs was determined in vitro by assessing embryoid body formation and performing immunofluorescence staining for endoderm (AFP), ectoderm (OTX2) and mesoderm (SMA) markers. The removal of Sendai virus vectors and transgenes (SeV, KOS, cMyc, and Klf4) was confirmed with quantitative PCR (qPCR). The absence of mycoplasma was analysed using conventional PCR (Venor GeM Classic Mycoplasma Detection Kit, Minerva Biolab). The karyotypes of the iPSC lines were analysed using the G-banding method at Fimlab Laboratoriot Ltd (Tampere, Finland). The genetic identities of the PBMCs and iPSCs were confirmed via a short tandem repeat (STR) analysis (GenePrint 24 system, Promega) at the Institute for Molecular Medicine Finland FIMM Technology Centre (University of Helsinki, Finland).

### Differentiation of iMGLs

MS (*n* = 6) and HC (*n* = 7) iPSCs were differentiated into iMGLs according to the protocol reported by Konttinen et al. [37], with light modifications. On Day 0, iPSCs were seeded at a density of 6,200–29,000 cells/cm^2^ on Matrigel (Corning)-coated dishes and cultured under low-oxygen conditions (5% O_2_, 5% CO_2_, 37 °C) until Day 4. On Days 0 and 1, the cells were maintained in E8 flex -medium containing 5 ng/ml BMP4, 25 ng/ml activin A (both from Peprotech) and 1 µM CHIR 99,021 (Axon). The medium contained ROCKi for the first two days of culture (Day 0: 10 µM and Day 1: 1 µM). From Day 2 until Day 8, the cells were cultured in basal medium containing DMEM/F-12 without glutamine, 1X GlutaMAX, 543 mg/l sodium bicarbonate (all from Thermo Fisher Scientific), 14 µg/l sodium selenite, 64 mg/l L-ascorbic acid (both from Sigma–Aldrich) and 0.5% penicillin/streptomycin (P/S). On Days 2 and 3, the basal medium was supplemented with 100 ng/ml FGF2, 50 ng/ml VEGF (both from Peprotech), 10 µM SB431542 and 5 µg/ml insulin (both from Sigma–Aldrich). From Day 4 onwards, the cells were maintained in a normoxic incubator. From Day 4 until Day 8, the basal medium was supplemented with 50 ng/ml FGF2, 50 ng/ml VEGF, 50 ng/ml TPO, 50 ng/ml IL-6, 10 ng/ml SCF, 10 ng/ml IL-3 (all from Peprotech) and 5 µg/ml insulin (Sigma-Aldrich) and changed daily. On Day 8, floating erythromyeloid progenitor cells (EMPs) were harvested and plated at a density of 64,000 cells/cm^2^ in ultralow attachment dishes (Corning). Cultures were maintained in basal medium containing Iscove′s modified Dulbecco′s medium (IMDM, Thermo Fisher Scientific), 10% heat-inactivated fetal bovine serum (FBS, Sigma–Aldrich) and 0.5% P/S supplemented with 5 ng/ml MCSF, 100 ng/ml IL-34 (both from Peprotech) and 5 µg/ml insulin. Beginning on Day 10, the medium supplemented with 10 ng/ml MCSF and 10 ng/ml IL-34 was changed every other day until the final plating on Day 16, when the cells were plated for experiments, after which 50% of the medium was changed daily. The experiments included immunocytochemistry, RNA-seq, qPCR, western blot, secretome profiling and phagocytosis assays, and they were performed on Days 21–23 in the basal state or after inflammatory treatments.

### Inflammatory treatments

The iMGLs were stimulated with lipopolysaccharide (LPS 0111:B4, 20 ng/ml, Sigma–Aldrich), interferon-γ (IFN-γ, 20 ng/ml, Peprotech), or a combination of LPS and IFN-γ (20 ng/ml for both) on Day 21 or 22. The iMGLs were stimulated for 45 min for NF-κB analyses and for 24 h for RNA-seq, qPCR, secretion and phagocytosis assays. FBS starvation was performed 24 h prior phagocytosis and secretion assays simultaneously with inflammatory stimulation. Each differentiation included both healthy and MS iMGL lines, and the results consist of multiple independent differentiations as described in the figure legends.

### RNA sequencing and analysis

The iMGLs were cultured on 6-well tissue-culture treated plates at a density of 400,000 cells/well and stimulated with LPS for 24 h. RNA was extracted from vehicle and LPS-treated iMGLs with a NucleoSpin RNA Kit (Macherey-Nagel) according to the manufacturer’s protocol, and the samples from two wells were pooled. RNA integrity was analysed with a DNF-471 RNA Kit (15 nt) and Fragment Analyzer (both from Agilent) according to the manufacturer’s instructions. Libraries were prepared with a CORALL Total RNA-Seq V2 Library Prep Kit with RiboCop (Lexogen). The quality of the prepared cDNA libraries was analysed with a High Sensitivity DNA Analysis Kit (Agilent). Sequencing was performed using an Illumina NextSeq500 sequencer with an Illumina NextSeq 500/550 High Output Kit v2.5, 75 cycles -kit (both from Illumina).

### Analysis of RNA sequencing data

The bulk RNA sequencing dataset was analysed using a series of established bioinformatic techniques. The raw count data were obtained with the nf-core rnaseq pipeline [52]. The raw count data were initially prepared by removing low-quality reads and normalizing by library size using the trimmed mean of M-values (TMM) method implemented in the edgeR package [53]. Quality control was performed by employing the similarity measure implemented in the StellarPath package [54], and the result was visualized through multidimensional scaling (MDS) to identify potential batch effects and outliers. This analysis revealed that the cell line was a significant confounding factor influencing the samples’ expression profiles. Therefore, cell line information was included as a covariate in the downstream differential expression analysis. Gene set variation analysis (GSVA) was performed using single-cell-specific markers derived from PanglaoDB [55] to identify the cell types contributing to each sample. The ssGSEA method was chosen for GSVA to calculate enrichment scores representing the degree of activation of each cell type-specific gene set in each sample. The same operation was also performed to compare our samples with the samples described by Abud et al. [38]. Subsequently, we also performed this task with the markers reported by Absinta et al. [20] to determine the type of microglia present in our samples. The differential expression analysis was then conducted using the limma package [56] with a contrast matrix designed to compare specific conditions of interest. This analysis aimed to identify genes whose expression levels were significantly altered between the compared conditions. Finally, a pathway analysis was performed using clusterProfiler [57] and msigdbr [58] to identify enriched pathways and biological processes associated with the observed changes in gene expression. This step provided insights into the biological functions and pathways affected by the experimental conditions. For the differential expression (DE) analysis and pathway analysis, we corrected the p-values for multiple testing with the Benjamini-Hochberg (BH) procedure.

Raw data from Absinta et al. [20] were downloaded from GSE180759, including count matrices and sample metadata. Only immune cells were retained for analysis. Marker genes for different immune cell types were obtained from Supplementary File 4 of the Absinta et al. publication. The analysis followed the Scanpy [59] workflow. Quality control was performed by removing cells with abnormally high expression of mitochondrial and ribosomal genes, as well as cells with an outlying number of counts and genes (defined as values lower or higher than three times the z-score). The count matrix was normalized using Pearson residuals [60]. Principal component analysis was conducted using the published marker genes as the most informative features. The 500 nearest neighbors were computed based on cosine distance, and Leiden clustering was applied. Clusters were annotated using Absinta et al.’s published marker genes. Differential expression analysis was performed between immune subtypes and between sample classes within each subtype using the Wilcoxon non-parametric test. Finally, pathway enrichment analysis was conducted using an over-representation test. Enrichr [61] was queried via the gseapy [62] package to identify significantly enriched pathways.

### Immunocytochemistry

iMGLs were seeded on tissue-culture treated 96-well plates at a density of 15,000 cells/well and fixed with 4% paraformaldehyde in phosphate-buffered saline (PBS) for 15 min. The cells were blocked with 10% normal donkey serum (NDS), 0.1% Triton X-100, and 1% bovine serum albumin (BSA) in PBS for 45 min at room temperature (RT). Primary antibodies were incubated with the cells in a solution containing 1% NDS, 0.1% Triton X-100, and 1% BSA in PBS overnight at 4 °C. The following primary antibodies were used: Iba1 (rabbit, 1:500; 019–19741; FujiFilm Wako), P2RY12 (rabbit, 1:125; HPA014518; Sigma-Aldrich), TMEM119 (rabbit, 1:100; ab185333; Abcam) and NF-κB p65 (rabbit, 1:400; D14E12; Cell Signaling Technology). The cells were incubated with an Alexa Fluor 488-conjugated donkey anti-rabbit secondary antibody (1:400; A21206; Thermo Fisher) diluted in 1% BSA in PBS for 1 h at RT. The stained cells were mounted with ProLong™ Gold Antifade Mountant with DAPI (Thermo Fisher Scientific) and imaged with an Olympus IX51 fluorescence microscope equipped with a Hamamatsu ORCA-Flash4.0 LT + sCMOS camera (type C11440-42U30). Both Iba1-positive iMGLs and NF-κB p65 nuclear translocation were quantified with CellProfiler (v4.2.1) and CellProfiler Analyst (v3.0.4) [63]. The NF-κB p65 analysis included three images per well and three wells per stimulation group. Images with low cell numbers (≤ 10 cells) were excluded from the analysis.

### qPCR

iMGLs were cultured on 6-well tissue culture-treated plates at a density of 400,000 cells/well. RNA was extracted from vehicle- and LPS-stimulated iMGLs after 24 h of treatment with a NucleoSpin RNA Kit (Macherey-Nagel) according to the manufacturer’s protocol. The RNA was reverse transcribed to cDNA with a High-Capacity cDNA Reverse Transcription Kit (Thermo Fisher Scientific). The utilized primers are listed in Supplementary Table 2. The mRNA expression levels were determined with TaqMan assays utilizing an ABI QuantStudio 12 K Flex Real-Time PCR System (Thermo Fisher Scientific). The data were analysed using the ΔΔCt method, with *GAPDH* (Hs99999905_ m1) serving as an endogenous control, and the data were normalized to those of the HC1 vehicle sample.

### Western blot analysis

iMGLs were cultured on 6-well tissue culture-treated plates at a density of 400,000 cells/well and treated with vehicle or LPS. The cells were washed with ice-cold PBS, and two wells were pooled with lysis buffer containing 50 mM Tris-HCl (pH 7.5), 10% glycerol, 150 mM NaCl, 1 mM EDTA, 50 mM NaF and 1% Triton X-100 (Sigma-Aldrich) on ice. Lysis buffer was supplemented with protease and phosphate inhibitor cocktails (both from Bimake). Lysed cells were centrifuged at 20 000 × g for 20 min at 4 °C. Protein concentrations were determined using Pierce™ 660 nm Protein Assay Reagent (Thermo Fisher Scientific) and 9 µg–5 µg of protein was separated on 10% Mini-PROTEAN^®^ TGX™ Precast Gels (456–1033, Bio-Rad) in Tris–Glycine–SDS running buffer. Proteins were transferred onto Trans-Blot^®^ Turbo™ Mini PVDF Transfer membranes (#1704156, Bio-Rad) utilizing a Trans-Blot Turbo Transfer System (Bio-Rad). The membranes were blocked with 4% BSA in PBS for 1 h at RT, after which they were incubated with primary antibodies in 4% BSA in PBS overnight at 4 °C. The following primary antibodies were used: NF-κB p65 (1:1000, mouse, 6956; Cell Signaling Technology), phospho-NF-κB p65 (1:1000, rabbit, 3033; Cell Signaling Technology), IκBα (1:1000, mouse, 4814; Cell Signaling Technology) and β-actin (1:2000, mouse, sc-47778; Santa Cruz). Thereafter, the membranes were labelled with the secondary antibody in 4% BSA in PBS for 1 h at RT. The following secondary antibodies were used: IRDye^®^ 800CW donkey anti-mouse IgG (1:20 000, 926–32212) and IRDye^®^ 680RD donkey anti-rabbit IgG (1:20 000, 926–68073, both from LI-COR Biosciences). Proteins were detected with LI-COR Odyssey CLx imaging system, images were quantified with Image Studio software (both from LI-COR Biosciences), and protein levels were normalized to those of β-actin. Normalized protein levels were used to quantify the ratio of phospho-NF-κB p65 to NF-κB p65.

### Phagocytosis assay

iMGLs were cultured on 96-well tissue culture-treated plates at a density of 15,000 cells/well and treated with vehicle, LPS, IFN-γ, or LPS + IFN-γ. After 24 h, the phagocytic capacity of iMGL was studied using pHrodo™ Green Zymosan Bioparticles™ Conjugate for Phagocytosis (P35365, Thermo Fisher Scientific). pHrodo bioparticles were added to the cells at a concentration 100 µg/ml in Opti-MEM (Thermo Fisher Scientific) supplemented with MCSF and IL-34 (both 10 ng/ml) and incubated for 6 h. The nuclei were stained with Hoechst 33,342 at the end of the experiment (1:1000, Thermo Fisher Scientific). The cells were imaged (1 image/well, 6 wells/group) with an Olympus IX51 fluorescence microscope equipped with a Hamamatsu ORCA-Flash4.0 LT + sCMOS camera (type C11440-42U30). A Leica DMi8 inverted microscope was used for live-cell imaging every 20 min (5%CO_2_, 20% O_2_, 37 °C; 3 images/well, 6 wells/group) to obtain a time curve after incubation with the pHrodo bioparticles for 2–6 h. The intensity and area of green fluorescence and the number of nuclei were analysed with CellProfiler [63] (v4.2.1). For the phagocytosis inhibition assay, iMGLs were pretreated with the actin polymerization inhibitor Cytochalasin D (10 µg/ml, Sigma–Aldrich) for 30 min prior to the addition of pHrodo bioparticles and imaged at 2 h.

### Cytokine secretion

iMGLs were seeded on 96-well tissue culture-treated plates at a density of 15,000 cells/well. The culture medium was pooled from two wells (*n* = 3 samples/group in each experiment). The secretion of cytokines and chemokines (TNF-α, IL-6, IL-10, IL-1β, GM-CSF, CCL2, CXCL5, CXCL8, and CXCL10) in the vehicle-, LPS- or IFN-γ-treated iMGL cell culture medium was measured using a U-PLEX Custom Biomarker Group 1 (human) Assay (Meso Scale Diagnostics) according to the manufacturer’s instructions. The samples were run on a MESO QuickPlex SQ120 and analysed with DISCOVERY WORKBENCH^®^ software (v4.0) (Meso Scale Diagnostics).

### Statistical analysis

The normality of the data was analysed with the Shapiro–Wilk test. Normally distributed data were analysed with independent-sample t tests. Nonparametric Mann–Whitney U tests and Kruskal-Wallis tests were used for nonnormally distributed data. Bonferroni’s or Dunn’s post hoc test was used for multiple comparisons. A p value < 0.05 was considered to indicate statistical significance. Statistical analyses were performed with IMB SPSS Statistics software (version 29.0) and GraphPad Prism (version 10).

## Results

### Brain TSPO-PET imaging reveals innate immune cell activation in PwMS

**Fig. 1:**
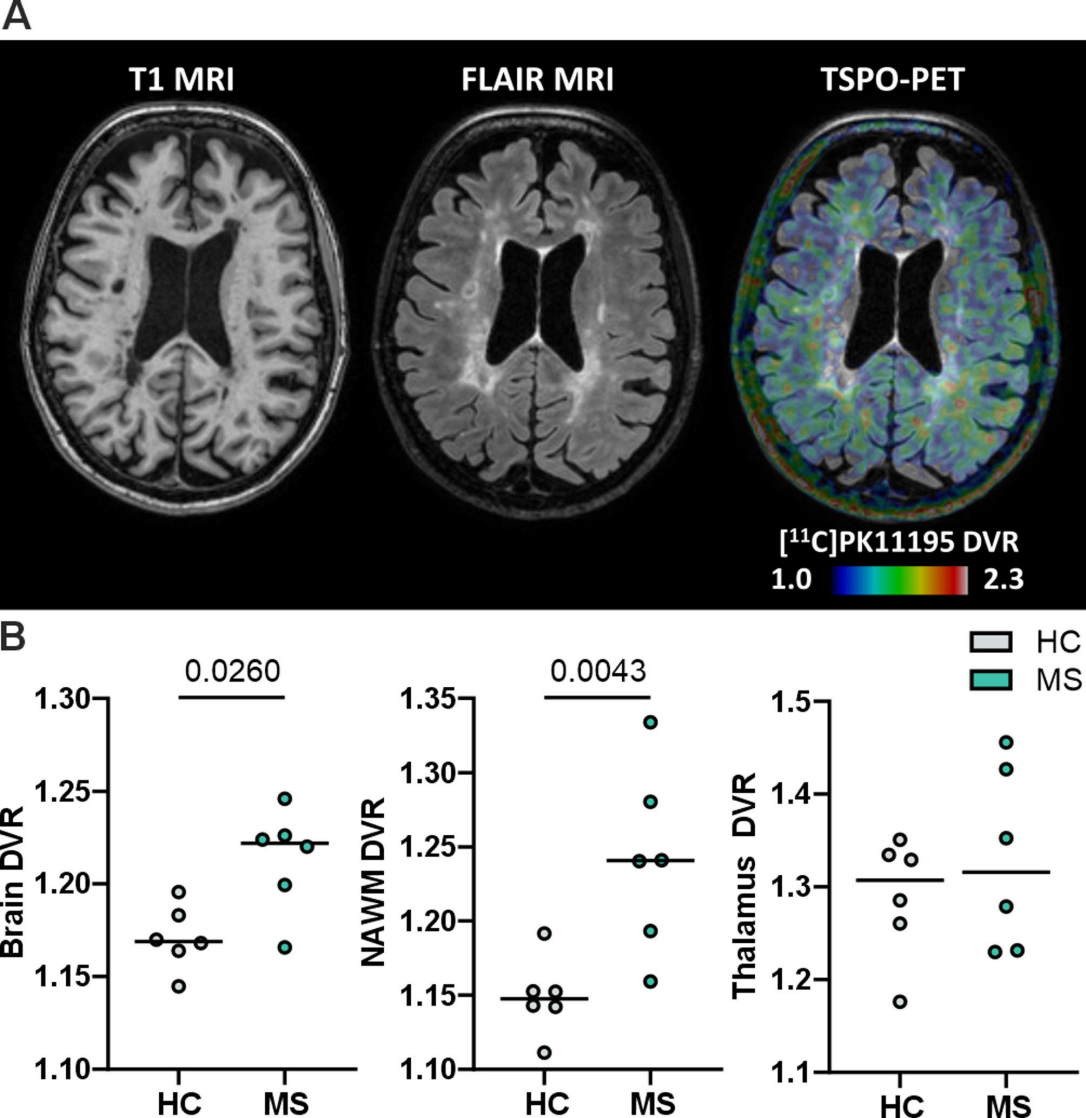
^11^C-PK11195-PET brain imaging of pwMS and HCs. **A** T1-weighted, FLAIR and TSPO-PET images of a patient with MS show a significant lesion load and increased innate immune cell activation, as evidenced by increased [^11^C]PK11195 binding in the NAWM and perilesional areas. The colour bar shows the dynamic range of the DVR in the PET image. **B** PET-measured brain DVR, NAWM DVR and thalamus DVR of the HCs (*n* = 6) and pwMS (*n* = 6) included in this study. The data are presented as single datapoints and medians. Significance was determined with the Mann–Whitney U test

We recruited six pwMS, including four patients with SPMS and two with RRMS disease. The clinical and radiological characteristics of the subjects are summarized in Table 1. The median disease duration was 16.9 (min–max 12.9–28.4) years representing generally a more advanced disease state. At the time of imaging, the median Expanded Disability Status Scale (EDSS) score was 3.5 (range 2.5–7.5). Half of the patients were untreated at the time of imaging or sampling. The patients were imaged with conventional MRI and PET (Fig. 1A), and the results were compared with those of age- and sex-matched healthy control subjects. MRI scans of the pwMS showed ongoing tissue damage and inflammation, with median (range) T1 and T2-detected lesion loads of 10.5 (0.1–28.1) and 16.6 (0.3–43.3), respectively (Table 1). Compared with HCs, brain volumetric measures indicated that pwMS presented with brain atrophy, as evidenced by significantly lower whole-brain and thalamic volumes but no changes in NAWM or cortical grey matter (GM) volumes (Table 1). PET imaging with the TSPO-binding radioligand [^11^C]PK11195 [44] was performed to evaluate ongoing innate immune cell activation. The distribution volume ratio (DVR), which is used to quantify TSPO binding, indicated significantly greater innate immune cell activation within the whole brain and NAWM of the pwMS than in HCs (1.222 vs. 1.169, median, *p* = 0.026 and 1.241 vs. 1.148, *p* = 0.0043), but no changes were observed in the thalamic region (Fig. 1B). These findings highlight ongoing innate immune cell activation and its potential role in the pathology of MS.

**Table 1:** Demographic, clinical and radiological characteristics of study subjects

Variable	HC	MS	MS vs. HC *p* value
n	6	6	
Sex, n			
Females	6	6	
Age	50.0 (44.7–60.1)	48.6 (45.7–56.7)	
Disease type			
RRMS		2	
SPMS		4	
Duration, years		16.9 (12.9–28.4)	
EDSS		3.5 (2.5–7.5)	
MSSS		3.7 (1.2–8.1)	
ARR		0.3 (0.2–0.8)	
Treatment			
Untreated		3	
Interferon-β		1	
Dimethylfumarate		1	
Glatiramer acetate		1	
MRI measures (volumes, cm^3^)			
T1		10.5 (0.1–28.1)	
T2		16.6 (0.3–43.3)	
Whole-brain (PF)	0.868 (0.836–0.916)	0.803 (0.769–0.879)	**0.041**
NAWM (PF)	0.354 (0.325–0.439)	0.309 (0.281–0.381)	0.132
GM cortex (PF)	0.333 (0.308–0.348)	0.305 (0.281–0.328)	0.065
Thalamus (PF)	0.012 (0.011–0.015)	0.009 (0.008–0.012)	**0.013**

Data are expressed as median (min–max) unless specified otherwise

Mann-Whitney U-test was used to compare MS vs. HC

Here, *p* < 0.05 is considered significant and values are bolded

Abbreviations: *HC* healthy control, *MS* multiple sclerosis, *RRMS* relapsing-remitting multiple sclerosis, *SPMS* secondary progressive multiple sclerosis, *EDSS* Expanded Disease Status Scale, *MSSS* Multiple Sclerosis Status Scale, *ARR* annualized relapse rate, *PF* parenchymal fraction, *NAWM* normal-appearing white matter, *GM* grey matter

### Differentiated MS iMGLs exhibit microglial features, but downregulate homeostatic markers

To investigate the MS-specific microglial phenotype and elucidate the potential disease mechanisms of microglial activation, we generated iPSC lines from these six pwMS. The iPSCs were successfully generated from PBMCs using Sendai virus reprogramming, and newly established iPSC lines were characterized based on their expression of pluripotency markers and trilineage differentiation capacity (Supplementary Fig. 1). A quality control assessment confirmed that the iPSC lines were free of viral transgenes and mycoplasma (Supplementary Fig. 2). The iPSCs presented a normal karyotype and correct cell line identity, as verified via short tandem repeat (STR) analysis (Supplementary Fig. 2). For comparison, seven iPSC lines derived from healthy controls were also utilized (Supplementary Table 1).

**Fig. 2:**
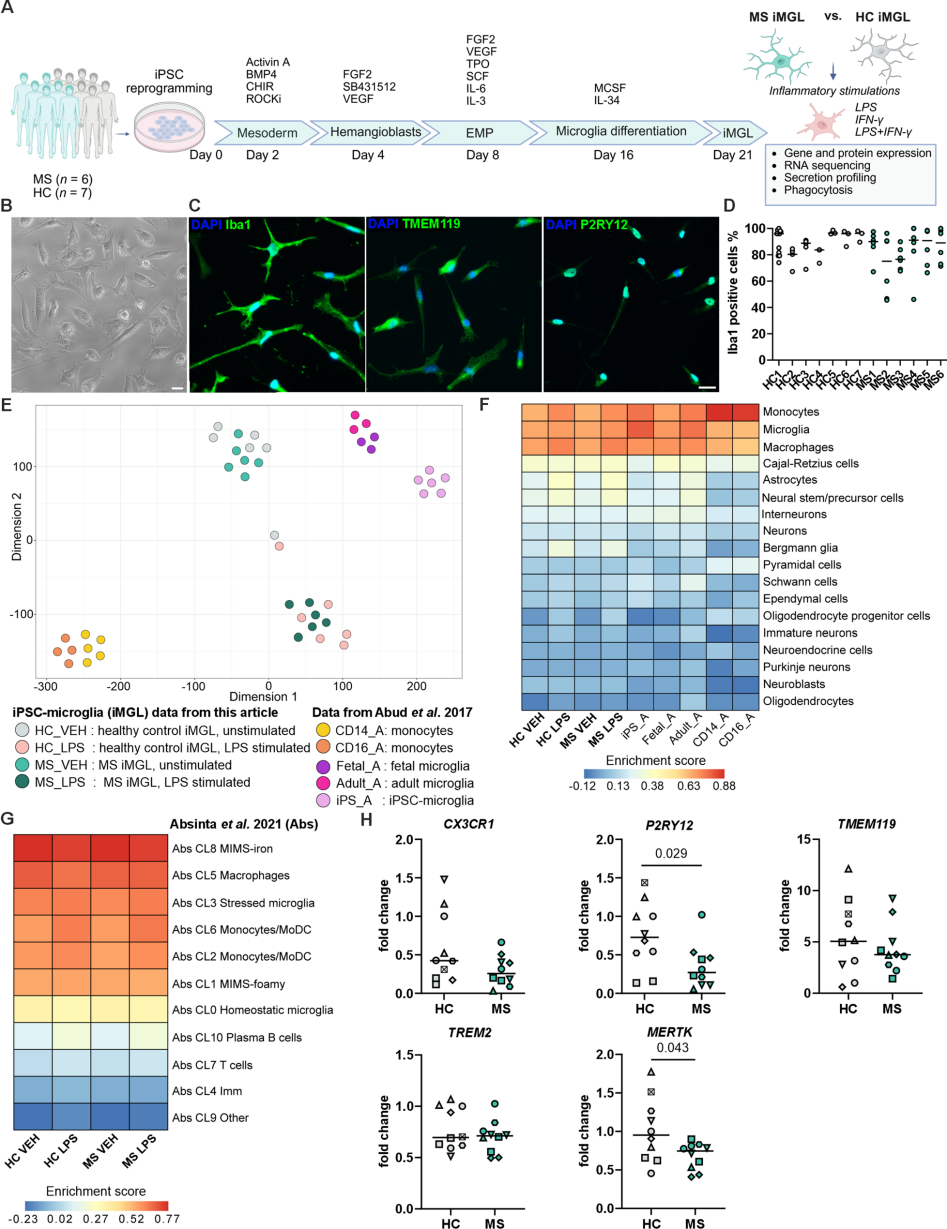
Generation and characterization of HC and MS iPSC-derived iMGLs. **A** Schematic overview of iMGL differentiation through erythromyeloid progenitors (EMP) and the experimental setup performed on HC and MS iMGLs. Representative images of **B** live cells and **C** immunofluorescence staining for Iba1, TMEM119 and P2RY12. Scale bar = 20 μm. **D** Percentages of Iba1-positive cells in immunofluorescence images, *n* = 3–12 images, with 1–4 independent differentiations per cell line. The data are presented as single datapoints and medians. **E** T-distributed stochastic neighbor embedding (t-SNE) plot shows similarity between expression profiles of vehicle- and LPS-stimulated HC and MS iMGLs, and iPSC-, fetal- and adult-derived microglia and monocytes from the Abud et al. [38] dataset. **F** Heatmap shows the results of the cell type enrichment analysis (PanglaoDB) [55] of our iMGL classes and Abud et al. [38] profiles. **G** Heatmap shows cell type enrichment analysis of our iMGLs, with the cell signature from the single-nucleus RNA sequencing clusters reported by Absinta et al. [20]. **H** qPCR analysis of microglial signature genes in HC and MS iMGLs. *n* = 10 differentiation batches, 7 HC cell lines and 6 MS cell lines, with 1–2 independent differentiations. The data are presented as mean values for individual iPSC lines and their independent differentiations. Symbol coding for iPSC lines is shown in Supplementary Table 1. Mann–Whitney U test. Panel A was created in BioRender. Hagman, S. (2025) https://BioRender.com/ql6t28z

For the differentiation of iMGLs we employed a previously published protocol by Konttinen and colleagues [37] that generates microglia-like cells by mimicking *bona fide* microglia originating from the yolk sac (Fig. 2A). All six MS and seven HC iPSC lines differentiated into iMGLs and presented a typical ramified morphology (Fig. 2B; Supplementary Fig. 3 A). Immunofluorescence staining revealed a high percentage of ionized calcium-binding adapter molecule 1 (Iba1) expression in iMGLs, with median values ranging from 75% to 96% across the lines, and showed ubiquitous expression of the microglia-specific proteins transmembrane protein 119 (TMEM119) and the purinergic receptor P2RY12 (Fig. 2C and D, and Supplementary Fig. 3B and C).

The microglial identity of differentiated cells was further confirmed via an RNA-seq analysis of four HC and six MS iPSC lines, including technical replicates for two of the HC lines. We challenged the iMGLs by exposing them to lipopolysaccharide (LPS), a common inflammatory stimulant used to activate microglia, and included LPS-stimulated iMGLs in our dataset. Multidimensional scaling analysis of gene expression profiles showed a clear separation between vehicle- and LPS-treated iMGLs (Fig. 2E). A comparative analysis with the publicly available dataset from Abud et al. [38] showed that our iMGLs closely resembled the microglial cells in their dataset while diverging from CD14^+^ and CD16^+^ monocytes (Fig. 2E). This finding was further validated by a cell type signature analysis, which showed that the expression profiles of our iMGLs were enriched for markers representative of microglia (Fig. 2F). Next, we used snRNA-seq dataset from immune cells of the chronic active MS lesions by Absinta et al. [20] to assess which type of microglial cell population determined the transcription of our iMGLs` genes. This analysis found that the iMGL is mainly enriched by “microglia inflamed in MS-iron” (MIMS-iron) located at the edge of chronic active lesions (Fig. 2G).

Microglial signature genes were further examined, revealing that iMGLs expressed *CX3CR1*, *P2RY12*, *TMEM119*, *TREM2*, and *MERTK* under basal conditions (Supplementary Fig. 4). Upon LPS treatment, these markers were downregulated, except for *MERTK* and *TMEM119*. Notably, RNA-seq analysis revealed a statistically significant reduction in the expression of key homeostatic marker *P2RY12* in MS iMGLs compared to HC iMGLs under basal conditions (Supplementary Fig. 4). This finding was further validated by qPCR for *P2RY12*, and *MERTK* also showed reduced expression (Fig. 2H).

Collectively, our data indicate that the HC and MS iMGLs present the characteristic phenotypic traits of human microglia and transcriptional traits of MS-associated microglia. However, MS iMGLs showed loss of homeostatic markers.

### Proinflammatory stimulation demonstrates the immune competence of the iMGLs

**Fig. 3:**
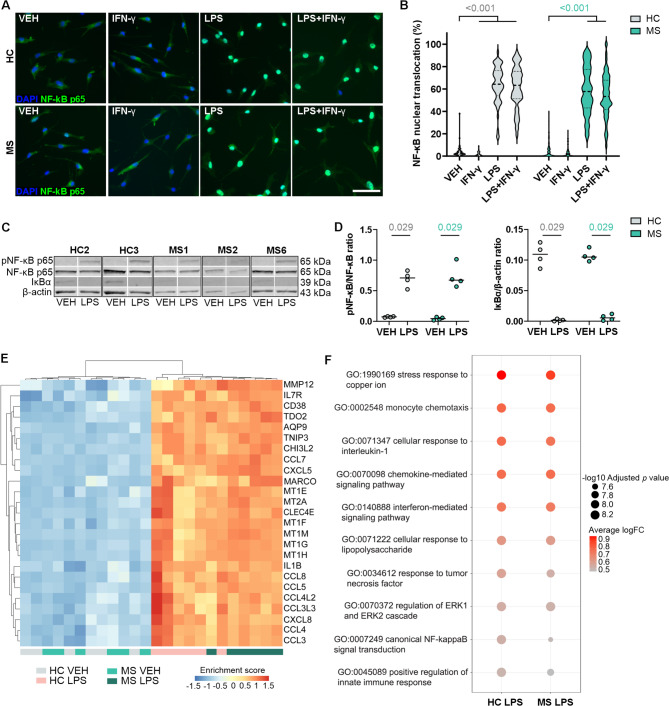
NF-κB activation and induction of inflammatory marker expression in LPS-treated iMGL. **A** Representative images of immunofluorescence staining for NF-κB p65 in vehicle-, IFN-γ-, LPS- or IFN-γ + LPS-stimulated HC and MS iMGLs. Scale bar = 50 μm. **B** Violin plot showing the quantification of the nuclear localization of NF-κB p65 from the immunofluorescence images. *n* = 54–118 images, 4 HC cell lines and 6 MS cell lines, with 2–3 independent differentiations. Mann–Whitney U test. The Bonferroni post hoc correction was used for multiple comparisons. **C** Representative western blots showing the levels of phospho-NF-κB p65, NF-κB p65, IκBα and β-actin as a loading control from vehicle- and LPS-treated (45 min, 20 ng/ml) HC and MS iMGLs. **D** Quantification of the phospho-NF-κB p65 and NF-κB p6 ratios and IκBα levels normalized to β-actin. *n* = 4 wells, 2 HC cell lines and 3 MS cell lines, with 1–2 independent differentiations, each with 1–2 samples. The data are presented as single datapoints and medians. Mann–Whitney U test. See also Supplementary Fig. 7. **E** Heatmap of the hierarchical clustering analysis showing the top 20 shared DEGs with the highest logFC values in the LPS treatment group compared with the vehicle group. The colour corresponds to the enrichment score. **F** Dot plot depicting the selected shared (gseGO) terms from DEGs of LPS treatment. The size depicts the significance level, and the colour scale depicts the average logFC

The transcription factor nuclear factor kappa-B (NF-κB) plays a central role in the pathogenesis of MS, mediating the activation of microglia [64]. We therefore wanted to test the immune competence and activation of this regulator of inflammation in iMGLs after stimulation with the proinflammatory factors IFN-γ (20 ng/ml), LPS (20 ng/ml) or their combination. Both HC and MS iMGLs rapidly responded to LPS, as evidenced by the nuclear translocation of NF-κB p65 after 15 min of exposure and a further increase after 45 min (Supplementary Fig. 5). The quantification of immunofluorescence staining after 45 min revealed NF-κB activation in a significant portion of iMGLs following LPS stimulation compared to vehicle-treated control cells (Fig. 3A and B). Compared with LPS alone, synergistic stimulation with LPS and IFN-γ led to similar levels of NF-κB activation in iMGLs. IFN-γ alone did not activate NF-κB in iMGLs. However, IFN-γ treatment altered the morphology, expression of microglial marker genes, and release of proinflammatory cytokines, confirming that iMGLs have the capacity to respond to several stimuli (Supplementary Fig. 6).

NF-κB activation was further investigated using western blotting, which allows an analysis of the phosphorylation of serine 536 on NF-κB p65 and the degradation of the inhibitory protein IκBa. As expected, quantification showed detection of phosphorylated NF-κB p65 after 45 min of LPS stimulation and, in contrast, IκBa levels were reduced, both of which are indicative of NF-κB pathway activation (Fig. 3C and D and Supplementary Figs. 7 and 8). However, no differences in the activation of NF-κB were observed between HC and MS iMGL.

Having shown the proinflammatory effect of LPS on NF-κB activation, we next sought to confirm the response at the transcriptome level after 24 h of treatment. The differential gene expression analysis revealed the greatest number of differentially expressed genes (DEGs; logFC ± 1 and adjusted *p* value < 0.05) between the LPS- and vehicle-treated groups, with 2,547 genes identified in HC iMGLs and 2,296 genes in MS iMGLs, confirming clear response to LPS (Supplementary Table 3). As anticipated, we observed the upregulation of numerous inflammatory genes in response to LPS treatment across different samples from both the HC and MS iMGL groups (Fig. 3E). Hierarchical clustering of the top shared DEGs revealed modules enriched in genes mediating inflammatory responses such as cytokines and chemokines and several metallothionine genes indicative of oxidative stress. Moreover, gene set enrichment analysis for Gene Ontology (gseGO) led to shared upregulation of processes related to the stress response to copper ion (*MT1G*,* MT1H*,* MT2A*, and *MT1M*), monocyte chemotaxis (*CCL7*,* CCL8*,* CCL5*,* CCL4*, and *CCL3*), chemokine-mediated signaling pathway (*CXCL8*,* CXCL5*, and *CCL7*) and canonical NF-κB signal transduction (*TNIP3*,* IL1B*, and *IL1A*) (Fig. 3F). The full list of DEGs and gseGO processes can be found in Supplementary Table 3.

Taken together, these findings indicate that the generated iMGLs can elicit specific responses to different inflammatory stimuli and that the LPS-response confirms the reproducible induction of inflammatory marker expression in iMGLs.

### RNA sequencing reveals the upregulation of transcripts associated with immune activation in MS iMGLs

**Fig. 4:**
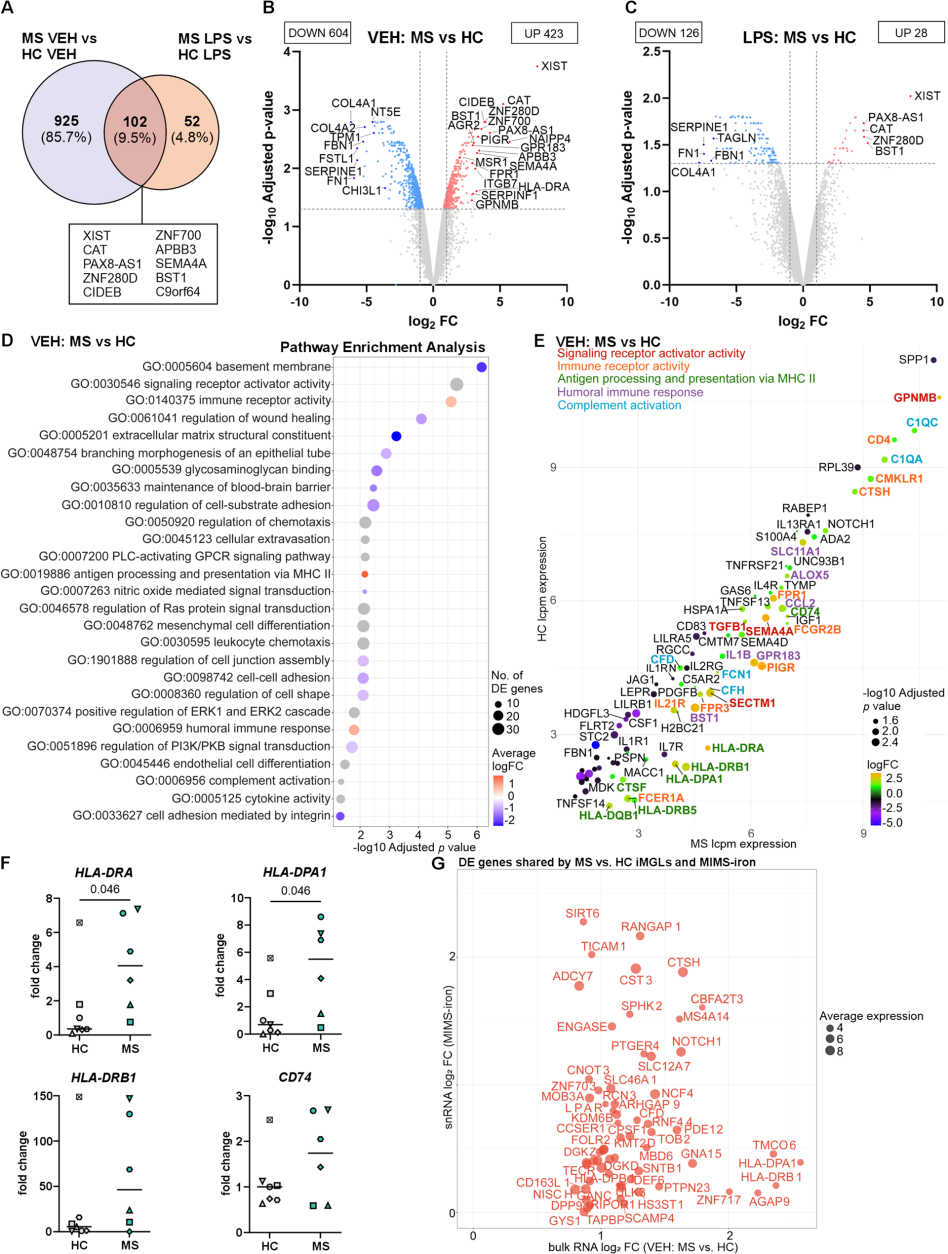
RNA sequencing analysis reveals a shift in the immune activation state of MS iMGLs. **A** Venn diagram displaying the number of differentially expressed genes (DEGs) between MS and HC iMGLs stimulated with vehicle or LPS for 24 h, with adjusted *p* values < 0.05 and logFC values > ± 1. Top overlapping upregulated DEGs between vehicle- and LPS-stimulated MS iMGLs. **B** Volcano plot showing DEGs identified between vehicle MS and HC iMGLs and **C** DEGs identified between LPS-stimulated MS and HC iMGLs with upregulated (red) and downregulated (blue) genes. The top and selected significant DEGs based on logFC are mentioned. **D** Dot plot displaying selected top Gene Ontology (GO) terms for vehicle MS vs. HC iMGLs. The colour scale indicates the average logFC and the size indicates the number of DEGs in the GO set. **E** Dot plot of the top DEGs enriched in selected GO terms for the comparison of vehicle-treated MS and HC iMGLs. Each dot represents a gene, the size indicates the significance level, genes are colour-coded by logFC, and selected gene labels are colour coded by pathways. *n* = 1–2 samples per cell line, 4 HC cell lines and 6 MS cell lines, with 1–2 independent differentiations. **F** qPCR analysis of *HLA-*genes of vehicle-treated HC and MS iMGLs. *n* = 6–7, 7 HC cell lines and 6 MS cell lines. The data are presented as mean values for individual iPSC lines and their independent differentiations. Symbol coding for iPSC lines is shown in Supplementary Table 1. Mann–Whitney U test. **G** Upregulated DE genes shared by our bulk RNA-seq MS iMGLs vs. HC iMGLs and published single-nucleus RNA-seq dataset of MIMS-iron microglial cells by Absinta et al. [20]. Dot size denotes average expression in bulk RNA-seq

Since microglia exhibit activated inflammatory phenotypes in rodent models of MS [21–23, 65] and in brain tissue samples from pwMS [20, 21, 27], we next performed a comparative whole-transcriptome RNA-seq analysis to determine whether MS iMGLs display cell-intrinsic alterations compared with HC iMGLs. We analysed all six MS lines and four HC lines under vehicle- and LPS-treated conditions. Remarkably, the DEG analysis confirmed the transcriptomic difference between MS and HC iMGLs in the basal state by identifying 1,027 DE genes (using a cut-off of logFC > ± 1 and adjusted p value < 0.05; Fig. 4A, and Supplementary Table 3). Furthermore, 154 genes were differentially expressed after LPS treatment between MS and HC iMGLs (Fig. 4A). Among these DEGs, 102 DEGs overlapped between the vehicle-treated group and the LPS-treated group (Fig. 4A).

DE genes observed between MS and HC iMGLs under vehicle- and LPS-treated conditions are displayed in volcano plots (Fig. 4B and C). Among the top DEGs in the vehicle-treated condition, novel genes, including *ZNF280D*,* CIDEB*,* ZNF700*,* APBB3*,* PIGR*, *ITGB7*,* BST1*,* AGR2* and *SERPINF1*, were upregulated. Moreover, our analysis revealed that several noncoding transcripts, including pseudogenes and long noncoding RNAs (*XIST*,* NAIPP4*, and *PAX8-AS1*), were differentially expressed in MS iMGLs. Other top DEGs in vehicle-treated MS iMGLs included genes previously described to be involved in MS pathology, such as *GPR183* (also known as *EBI2*) and *FPR1* [66–68], or those directly linked to MS microglia, such as *CAT*,* SEMA4A*,* HLA-DRA*,* HLA-DPA1*,* GPNMB*,* SLC11A1*,* CD74*,* MSR1*,* ALOX5*,* HSPA1A*, *C1QA* and *FCGR2B* [20, 21, 25, 27, 69–72]. Most of the top upregulated genes in the MS vs. HC iMGL comparison were shared between the vehicle- and LPS-treated groups (Fig. 4A-C). This small subset of dysregulated genes included *XIST*, *CAT*, *CIDEB*, *APBB3*, *SEMA4A* and *BST1* (Fig. 4A and Supplementary Table 3), which have been implicated in immune regulation and oxidative stress responses [70, 73].

We further elucidated the biological functions involved in MS and the GO enrichment analysis of MS iMGLs highlighted significantly upregulated genes associated with immune responses in the basal state. These terms included “immune receptor activity” (*PIGR*,* FPR1*,* IL21R*,* FPR3*,* FCER1A*,* CTSH*, and *FCGR2B)*, “antigen processing and presentation of exogenous peptide antigen via major histocompatibility complex (MHC) class II” (*HLA-DRA*,* HLA-DPA1*,* HLA-DRB1*,* HLA-DQB1*,* CD74*, and *HLA-DRB5*), “humoral immune response” (*BST1*,* GPR183*,* SLC11A1*,* ALOX5*,* CCL2*, and *IL1B*), and “complement activation” (*C1QA*,* FCN1*, and *C1QC*), among others (Fig. 4D). In addition, downregulated genes (including *COL4A1*,* FN1*,* SERPINE1*,* FSTL1*,* FBN1*,* COL4A2*,* TPM1*, and *CHI3L1*) were enriched for terms related to extracellular matrix (ECM) organization, wound healing, cell adhesion and the regulation of cell shape (Fig. 4B and D). Similarly, LPS stimulation mainly downregulated these ECM-associated pathways in MS iMGLs compared with HC iMGLs (Supplementary Table 3). A complete list of significant pathways identified with eGO is available in Supplementary Table 3.

Next, we investigated five of the deregulated immune process-related pathways and their associated genes that influence the microglial phenotype. We compared the expression levels, fold change values and adjusted *p* values of DEGs between vehicle-treated MS iMGLs and HC iMGLs to identify the most significant genes (Fig. 4E). Interestingly, we distinguished several of the novel top genes (*PIGR*,* BST1*, and *FPR3*) with immune functions and confirmed genes previously associated with MS pathology (*SEMA4A*,* HLA-DRA*,* HLA-DPA1*,* GPNMB*,* SLC11A1*,* CD74*,* ALOX5*,* HSPA1A*, *C1QA* and *FCGR2B*) that align with the active microglial state reported in various neurodegenerative diseases, including MS [20, 21, 25, 27, 69–72].

The expression of selected top genes from RNA-seq analyses was assessed using qPCR in all six MS lines and all seven HC lines under basal state (Supplementary Fig. 9). Interestingly, analysis confirmed significantly elevated expression of *HLA-DRA* (median FC 4.0 vs. 0.4, *p* = 0.046) and *HLA-DPA1* (median FC 5.5 vs. 0.7, *p* = 0.046) in MS iMGLs compared to the HC iMGLs (Fig. 4F). There was also a trend toward higher expression of *HLA-DRB1* (median FC 46.9 vs. 5.6, *p* > 0.05) and *CD74* (1.8 vs. 1.0, *p* > 0.05) in MS iMGLs compared to the HC iMGLs, although this did not reach statistical significance.

Finally, as our iMGL was mainly enriched by MS lesion-specific microglial cell population MIMS-iron (Fig. 2G), we further investigated how transcriptional differences in MS iMGLs relate to deregulations shown by this MS-associated microglial phenotype. We analysed the immune cells of the Absinta et al. snRNA-seq dataset [20] and compared the signature genes and pathways of MS lesion-specific microglia against the signatures of our MS iMGLs. This comparison revealed that shared genes, such as *HLA-DPA1*, *HLA-DRB1*, *PTGER4*, *CFD*, *NOTCH1*,* DEF6*, *GNA15* and *GRK*, were elevated (Fig. 4G), indicating enhanced antigen presentation and proinflammatory signaling. Upregulated pathways were predominantly immune-related, with enrichment in MHC class II antigen presentation and G-protein coupled receptor (GPCR) signaling (Supplementary Table 3). Conversely, downregulated pathways were related to ECM organization, including genes such as *ITGB1*, *COL4A2*, and *SERPINE1*.

In summary, these results reveal that MS iMGLs present intrinsic immune activation compared with HC iMGLs which is more evident in the basal state than after strong inflammatory stimulation with LPS. Furthermore, we identified the top DEGs and pathways related to antigen presentation, immune receptor activity and complement activation, indicating that increased microglial activation potentially plays a role in disease pathogenesis.

### Modulation of cytokine release in MS iMGLs

**Fig. 5:**
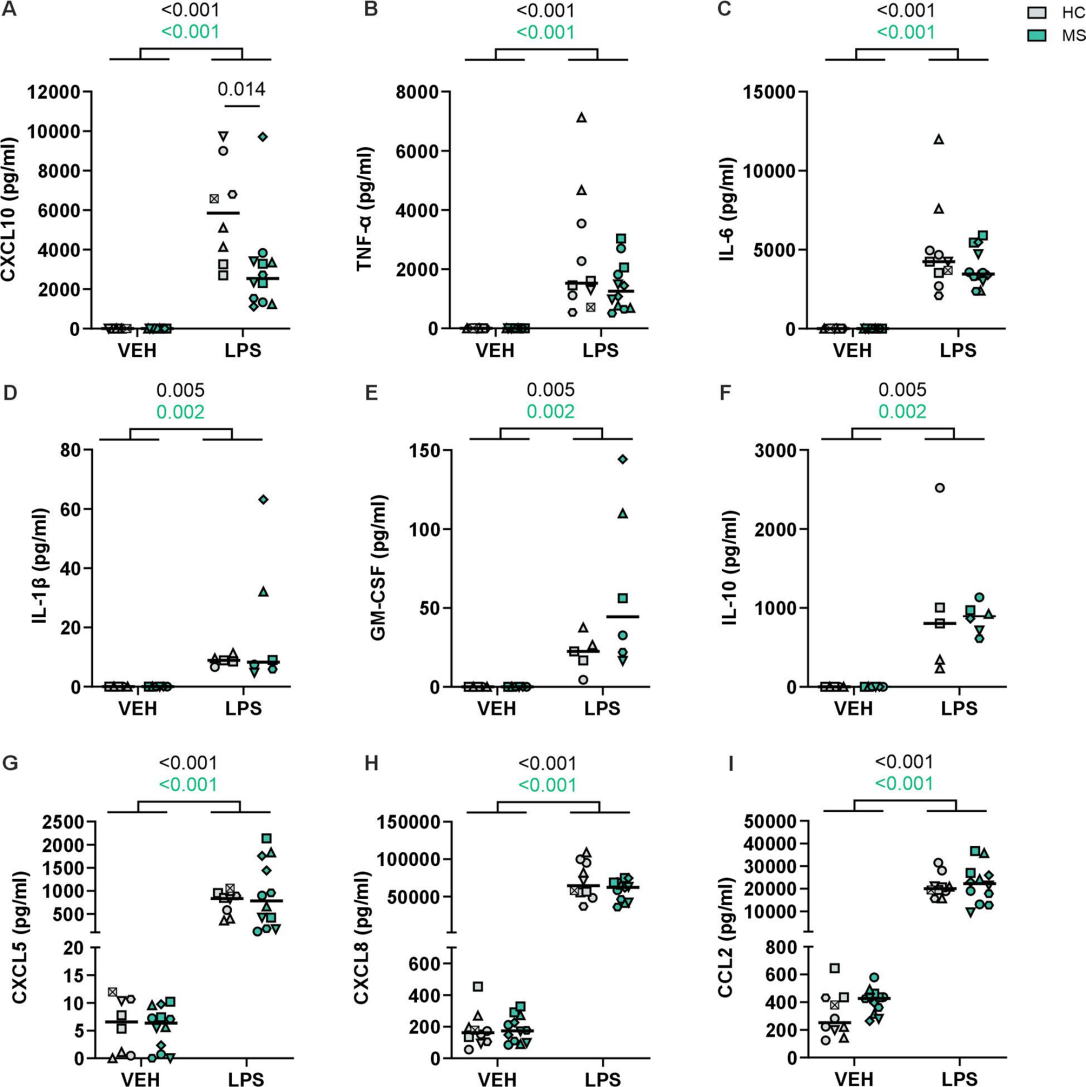
MS iMGLs exhibit altered cytokine release. Secretion of cytokines from HC and MS iMGLs stimulated with vehicle or LPS for 24 h. Multiplexed assay measuring **A** CXCL10, **B** TNF-α, **C** IL-6, **D** IL-1β, **E** GM-CSF, **F** IL-10, **G** CXCL5, **H** CXCL8, and **I** CCL2 levels in the media. *n* = 5–12, 3–6 HC cell lines and 6 MS cell lines, with 1–3 independent differentiations per cell line. The data are presented as mean values for individual iPSC lines and their independent differentiations. Symbol coding for iPSC lines is shown in Supplementary Table 1. Mann–Whitney U test

As several immune-related pathways were upregulated in MS iMGLs these observations prompted us to investigate the release of inflammatory factors, a hallmark of microglial activation [15]. The release of various inflammatory cytokines and chemokines in the culture medium was analysed with multiplex assays following 24 h of vehicle or LPS stimulation. In the basal state, the secreted levels of CXCL10, TNF-α, IL-6, IL-1β, GM-CSF and IL-10 were negligible or lower than the detection range (< 1 pg/ml), whereas the CXCL5 levels were low (< 12 pg/ml), and the CXCL8 and CCL2 levels were considerable (median 200–400 pg/ml) (Fig. 5). Although not statistically significant, MS iMGLs secreted more CCL2 in the basal state than HC iMGLs did (Fig. 5I), showing a pattern that was similar to the pattern of mRNA expression in the RNA-seq data (Fig. 4E).

LPS stimulation provoked a significant increase in the release of all measured cytokines and chemokines in both HC and MS iMGLs, indicating that LPS induced a switch from a surveying state to a proinflammatory microglial state. Compared with HC iMGLs, MS iMGLs produced significantly less CXCL10 (Fig. 5A). Concomitantly, MS iMGLs released higher levels of GM-CSF, although this did not reach statistical significance (Fig. 5E).

Thus, the generated iMGLs respond to the LPS stimulus, as expected, with increased cytokine and chemokine release. However, compared with HC iMGLs, MS iMGLs exhibit a modest alteration in cytokine secretion.

### MS iMGLs show enhanced phagocytic function

**Fig. 6:**
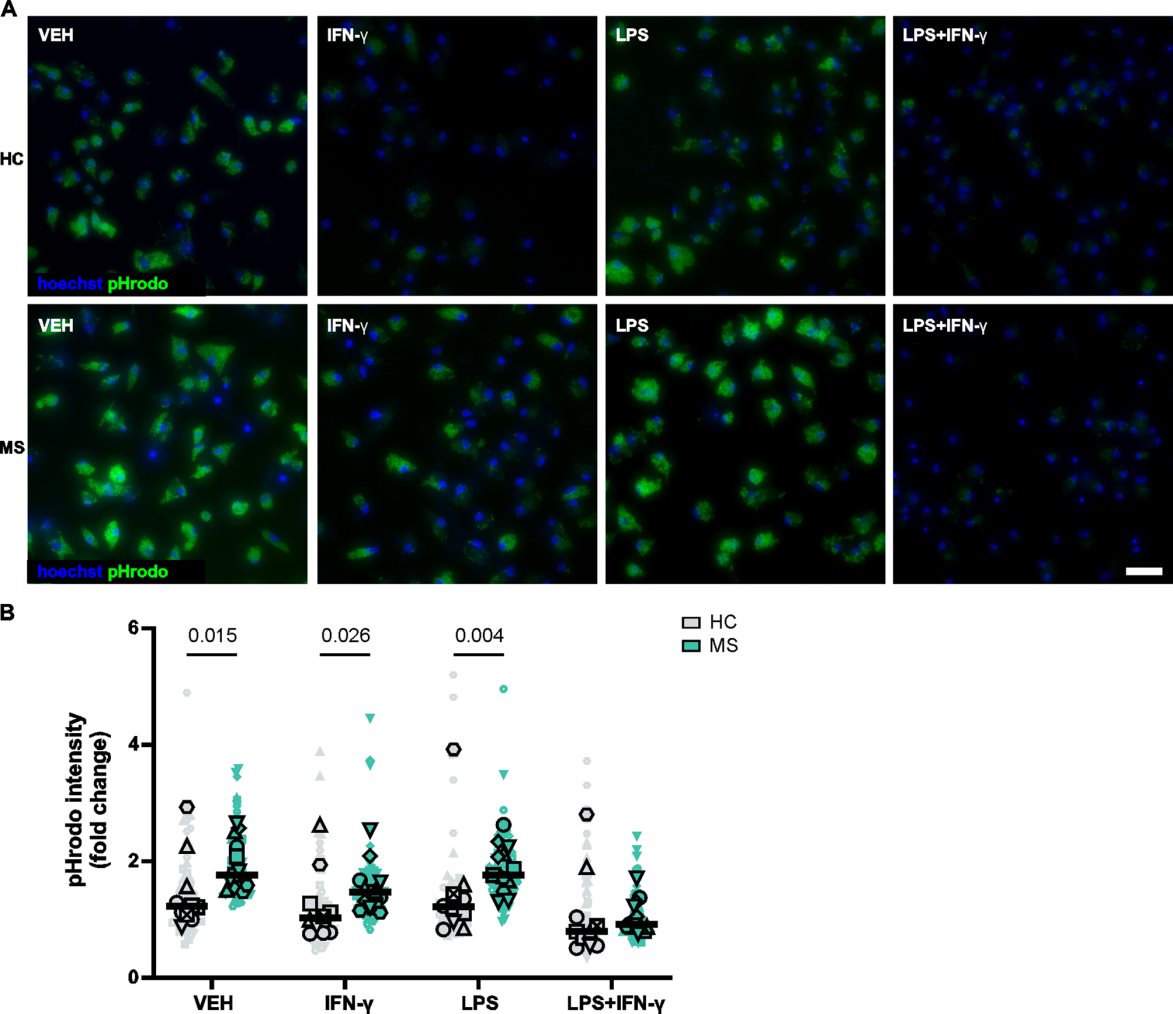
The phagocytic capacity of MS iMGLs is enhanced and modulated by inflammatory stimulation. **A** Representative fluorescence images of pHrodo zymosan bioparticles phagocytosed at 5 h after 24 h of vehicle, IFN-γ, LPS or IFN-γ + LPS stimulation in HC and MS iMGLs. Scale bar = 50 μm. **B** Quantified pHrodo intensity in HC and MS iMGL samples compared with the HC1 VEH sample. Each larger data point represents the mean value for an individual iPSC line derived from an independent differentiation. Small data points represent all measurements. Symbol coding for iPSC lines is shown in Supplementary Table 1. *n* = 10–13, 6 HC cell lines and 6 MS cell lines, with 1–3 differentiations per cell line. Mann–Whitney U test

Phagocytosis is a key function of microglia [16]; therefore, we explored whether MS iMGLs present functional changes in phagocytosis that are indicative of disease-promoting activity. Phagocytic function was evaluated using pHrodo zymosan A bioparticles and fluorescence imaging. We also tested the effects of proinflammatory pretreatment with IFN-γ, LPS or their combination on the phagocytosis of iMGLs. The spontaneous phagocytosis of the zymosan bioparticles was followed over 6 h (Supplementary Fig. 10 A), and cytochalasin D treatment served as a negative control (Supplementary Fig. 10B and C). We observed phagocytosis in both HC and MS iMGLs, which was further modulated by inflammatory stimulation (Fig. 6A). Pretreatment of iMGLs with IFN-γ, and particularly with the combination of LPS + IFN-γ, suppressed phagocytosis in many of the studied iMGL lines (Supplementary Fig. 10D). LPS pretreatment however, resulted in variable effects in phagocytosis across some of the HC and MS iMGL lines tested (Supplementary Fig. 10D). The most notable finding was that phagocytic function was significantly increased in MS iMGLs compared to HC iMGLs. While inflammatory stimulation modulated phagocytosis, MS iMGLs still exhibited significantly greater phagocytosis than HC iMGLs did, except under LPS + IFN-γ stimulation (Fig. 6B). Thus, altered or excessive microglial phagocytosis may contribute to multiple sclerosis pathology.

## Discussion

In this study, we investigated the intrinsic alterations of iPSC-based iMGLs derived from pwMS. We observed innate immune cell activation using TSPO-PET imaging from progressed pwMS compared with the controls and successfully derived MS-specific iMGLs from these patients. Transcriptomic data revealed that MS iMGLs upregulated numerous genes previously linked to MS pathology and pathways related to immune regulation, antigen presentation and complement activation. This result was further supported by key functional assays showing modulation of inflammatory cytokine secretion and increased phagocytic capacity.

In the context of MS, an increasing body of evidence indicates that the CNS compartmentalized inflammation driven by innate immune cells contributes to disease progression independently of relapse activity [7, 10, 14]. Both diffuse microglial activation in the NAWM and an increased chronic active lesion load, characterized by widespread microglial activation at the lesion edge, play significant roles in neurodegeneration [1, 7]. In the present study, increased innate immune cell activation was detected in pwMS using the TSPO-PET in the whole brain and the NAWM, which has been previously shown to predict later disease progression [14]. Therefore, we studied whether the intrinsic disease mechanisms of microglia can be recapitulated with iPSC-derived models. Here, iMGLs were successfully generated from these patients to introduce novel preclinical models of MS microglia. Although previous work by us and others using iPSC-derived glial and neuronal cells has shown that iPSCs provide a complementary platform to rodent models to study neuroinflammatory and neurodegenerative mechanisms in vitro [37, 74], to the best of our knowledge, the microglial phenotype has not been previously studied using iMGLs derived from pwMS.

First, our findings show that differentiated iMGLs closely correlate with the microglial signature observed in the previous studies of iPSC microglia, confirming the correct cell phenotype [38]. Additionally, we were able to show that our differentiated iMGL transcriptional profile is determined by the microglial cell type found in in vivo MS lesions, particularly the cluster identified as MIMS-iron at the chronic active lesion rim [20]. Although microglia cultured in vitro may not fully replicate the key transcriptional features observed in vivo and often inherently show inflammatory characteristics [75, 76], iMGLs have been shown to generate diverse transcriptional profiles under in vitro conditions [77], supporting their relevance as models for disease-associated microglial phenotypes. Interestingly, our data revealed the downregulation of the homeostatic microglial gene *P2RY12* in MS iMGLs in the basal state. Consistent with our observation, earlier studies of postmortem tissues from patients with MS have indicated that microglia lose their homeostatic characteristic expression of *P2RY12* [20, 24]. These findings strongly support the use of the MS iMGL cell model as a powerful tool for revealing the cell-autonomous microglial alterations in pwMS.

Here, the transcriptional signature of MS iMGLs revealed the most biologically relevant changes occurring in the basal state without an external inflammatory stimulus, including the detection of novel immune-related transcripts such as *PIGR*, *BST1* and *FPR3* [78, 79]. Interestingly, we also detected increased expression of the long noncoding RNA *XIST*, which has been recently described to contribute to inflammatory responses that potentially drive female-biased autoimmunity [80]. In addition, a previous snRNA-seq study of pwMS identified *XIST* among the top DEGs in a cluster annotated as macrophages at the chronic active lesion edge [20], suggesting that *XIST* may be an important regulator of microglia and macrophage activation. The observation that a subset of these genes remains persistently dysregulated in MS iMGLs following LPS stimulation suggests the MS iMGLs have a preactivated or primed state that is resistant to acute inflammatory stimulation. Several of the persistently dysregulated genes have been implicated in, for instance, immune regulation and oxidative stress responses [70, 73]. The recurrence of these genes across conditions and donors supports their biological relevance to MS pathogenesis. Future studies investigating genetic variants associated with MS risk and aligning them with differentially expressed genes could help clarify whether transcriptional signatures arise from genetic predisposition or are a consequence of cellular reprogramming.

Most importantly, at basal state MS iMGLs exhibited the upregulation of multiple genes previously reported to be expressed in MS microglia, such as *CAT*,* SEMA4A*,* HLA-DRA*,* HLA-DPA1*,* GPNMB*,* SLC11A1*,* CD74*,* MSR1*,* ALOX5*,* HSPA1A*, *C1QA* and *FCGR2B* [20, 21, 25, 27, 69–72]. Among these, glycoprotein *GPNMB* is a known regulator of microglial immune responses, is linked to the phagocytic microglial phenotype and has been suggested as a marker for lesion-associated microglia [29, 69, 81]. Transcriptomic differences between MS and HC iMGLs in the basal state emphasized the role of microglia in immunoregulation, including the positive enrichment of pathways associated with immune receptor activity, antigen presentation and complement activation, among other processes, with known implications in MS [20]. The increased expression of HLA-genes involved in antigen presentation, as observed in our RNA-seq data, was also verified using qPCR in MS iMGLs. This finding aligns with previous studies showing that in MS, activated microglia increase MHC molecules on their surface and can present antigens that activate T cells [20, 82]. In addition, MS iMGLs upregulate complement genes compared with HC iMGLs, which is consistent with the involvement of the complement cascade in microglial activation and microglia-mediated synapse loss observed in MS [83]. Comparison of our bulk RNA-seq data with published snRNA-seq data of microglia from MS lesions revealed shared genes involved in antigen presentation and immune signaling aligning their transcriptional profile with MS disease-associated microglial subtypes. Based on these findings, MS iMGLs present signs of cell-autonomous immune activation that may be key in CNS-confined MS pathology.

We examined these findings at the functional level and measured the release of inflammatory mediators from the culture media. The analysis showed increased trend in secretion of CCL2 from MS iMGLs, which was in accordance with our RNA-seq data, implying that CCL2 may contribute to microglial activation in the basal state. A previous study using scRNA-seq of microglia from early active pwMS identified a cluster containing preactivated microglia with elevated CCL2 levels [21]. CCL2 has an important function in the recruitment of lymphocytes to the CNS and is recognized as a key regulator of glial function and microglial activation [84]. Moreover, in our study, LPS treatment activated the key inflammatory signalling cascade NF-κB in iMGLs, leading to changes in the secretion of NF-κB targets [64], including inflammatory cytokines and chemokines. Specifically, the secretion of GM-CSF showed an increased trend following LPS challenge in MS iMGLs compared to HC iMGLs. Although GM-CSF plays an important role in microglial homeostasis, it also affects proliferation, antigen presentation, and phagocytosis [85]. In contrast to a previous study of MS iPSC-derived astrocytes showing increased levels of a specific set of NF-κB targets following activation [34], in our MS iMGLs, the levels of many of the tested cytokines and chemokines were unchanged, except for CXCL10, which was decreased compared with those in HC iMGLs. A plausible explanation for this reduced inflammatory response following LPS challenge is the dysregulated inflammatory signaling, as our RNA-seq data, decreased *P2RY12* expression and upregulation of CCL2 suggest that our MS iMGLs represent an already polarized immune state under basal conditions.

In MS, microglial phagocytosis plays a crucial role in the engulfment of myelin and clearance of cellular debris and contributes to synapse loss [25, 69, 83]. Here, we tested the phagocytic function of iMGLs and observed dampened phagocytosis after IFN-γ and a combination of LPS and IFN-γ treatment, similar as previously shown with iPSC-derived iMGLs [37, 86]. Still, we reported significantly enhanced phagocytosis both in the basal state and upon inflammatory challenge with LPS and IFN-γ in MS iMGLs compared to HC iMGLs. Many of the upregulated genes that were detected in our MS iMGLs have also been linked to phagocytosis in transcriptional studies of postmortem tissues from pwMS [20, 21, 25] suggesting that dysregulated phagocytosis contributes to MS pathology. Notably, a recent comprehensive analysis of microglial nodules from the NAWM revealed that activation by cytokines alongside phagocytosis of oxidized phospholipids likely contribute to a microglial phenotype that is predisposing to MS lesion formation [25]. The mechanisms underlying phagocytosis in MS warrant further investigation, particularly regarding the internalization and processing of myelin debris. In the current study, we used zymosan bioparticles, however, future studies incorporating MS-relevant myelin debris could provide deeper insights into how altered clearance of myelin debris may contribute to chronic inflammation.

Although the presented iPSC-derived MS microglial model allows us to elucidate cell type-specific disease mechanisms in a controlled setting, information on the effects of the complex three-dimensional cytoarchitecture and cell–cell interactions with other CNS or peripheral immune cells on microglial function is limited. Therefore, the development of more physiologically relevant cell models, such as the recently described glia-enriched organoid model [87] incorporating MS iMGLs, would be necessary. Furthermore, even though we performed a thorough characterization of the seven healthy control and six MS iMGL lines, adding more iPSC lines derived from patients presenting different disease types and levels of microglial activation, and other brain diseases would provide valuable insights into how the in vitro findings correlate with the in vivo observations. Although we revealed MS-specific iMGL phenotype in same patients who exhibited TSPO activity, it is important to acknowledge that while the TSPO-PET signal primarily reflects increased densities of activated microglia and macrophages, the determination of its exact cellular source remains challenging. However, recent work has shown association between TSPO-related innate immune cell activation and more rapid disease progression, using both a large neuropathological sample and a large cohort of TSPO-imaged pwMS demonstrating the clinical relevance of TSPO-PET imaging despite its cellular resolution limitations [88]. By including TSPO-PET imaging data from the same pwMS who donated iPSCs, we demonstrate a direct association between innate immune activation in vivo and adverse microglial phenotypes in vitro, underscoring the translational value of this work.

## Conclusion

In conclusion, these data demonstrate that microglia derived from pwMS exhibit cell-autonomous immune activation that may contribute to the neuroinflammatory milieu and pathological processes in MS. In this study, we demonstrate that MS iMGLs upregulate genes associated with MS pathology and immune regulation. Additionally, we describe that MS iMGLs alter the production of inflammatory factors and exhibit enhanced phagocytosis, which may contribute to a hyperactivated microglial state in MS. Therefore, these findings indicate that microglial activation in the MS context can be studied with iPSC-derived iMGLs, and these cells represent a unique platform for the identification and testing of novel treatment targets.

## Supplementary Information

Supplementary Material 1.

Supplementary Material 2.

## Data Availability

The data supporting the conclusions of this article are included within the article and its additional files. The data are available from the corresponding author upon reasonable request. The raw RNA-seq data are not publicly available due to their containing information that could compromise the privacy of research participants.

## Abbreviations

CNS: Central nervous system
DEG: Differentially expressed gene
DVR: Distribution volume ratio
ECM: Extracellular matrix
EDSS: Expanded disability status scale
EMP: Erythromyeloid progenitor cell
GWAS: Genome-wide association study
HC: Healthy control
IPSC: Induced pluripotent stem cell
IFN: Interferon
IL: Interleukin
LPS: Lipopolysaccharide
iMGL: Human induced pluripotent stem cell-derived microglia-like cell
MHC: Major histocompatibility complex
MIMS: Microglia inflamed in MS
MRI: Magnetic resonance imaging
MS: Multiple sclerosis
NAWM: Normal-appearing white matter
PBMC: Peripheral blood mononuclear cell
PET: Positron emission tomography
pwMS: patient with MS
RNA-seq: RNA sequencing
RRMS: Relapsing-remitting MS
SPMS: Secondary progressive MS
TNF: Tumor necrosis factor
TSPO: 18-kDa translocator protein
