# Supplementary material for: Microglia from patients with multiple sclerosis display a cell-autonomous immune activation state
Source: J Neuroinflammation. 2025 Oct 31;22:255. doi: 10.1186/s12974-025-03575-4 (PMC12577231; doi:10.1186/s12974-025-03575-4)
Supplement: Supplementary file 1 — Supplementary Material 1. [file 12974_2025_3575_MOESM1_ESM.pdf]

Supplementary information

Microglia from patients with multiple sclerosis display a cell-autonomous immune activation state

Tanja Hyvärinen[1,†], Johanna Tilvis[1,†], Luca Giudice[2], Iisa Tujula[1], Marjo Nylund[3,4,5,6], Sohvi Ohtonen[2], Flavia Scoyni[2,7], Henna Jäntti[2], Lassi Virtanen[1], Sara Pihlava[1], Roosa Kattelus[1], Heli Skottman[8], Susanna Narkilahti[9], Laura Airas[3,4,5,6], Tarja Malm[2], Sanna Hagman[1]

[†]These authors contributed equally to this work.

Supplementary Figures

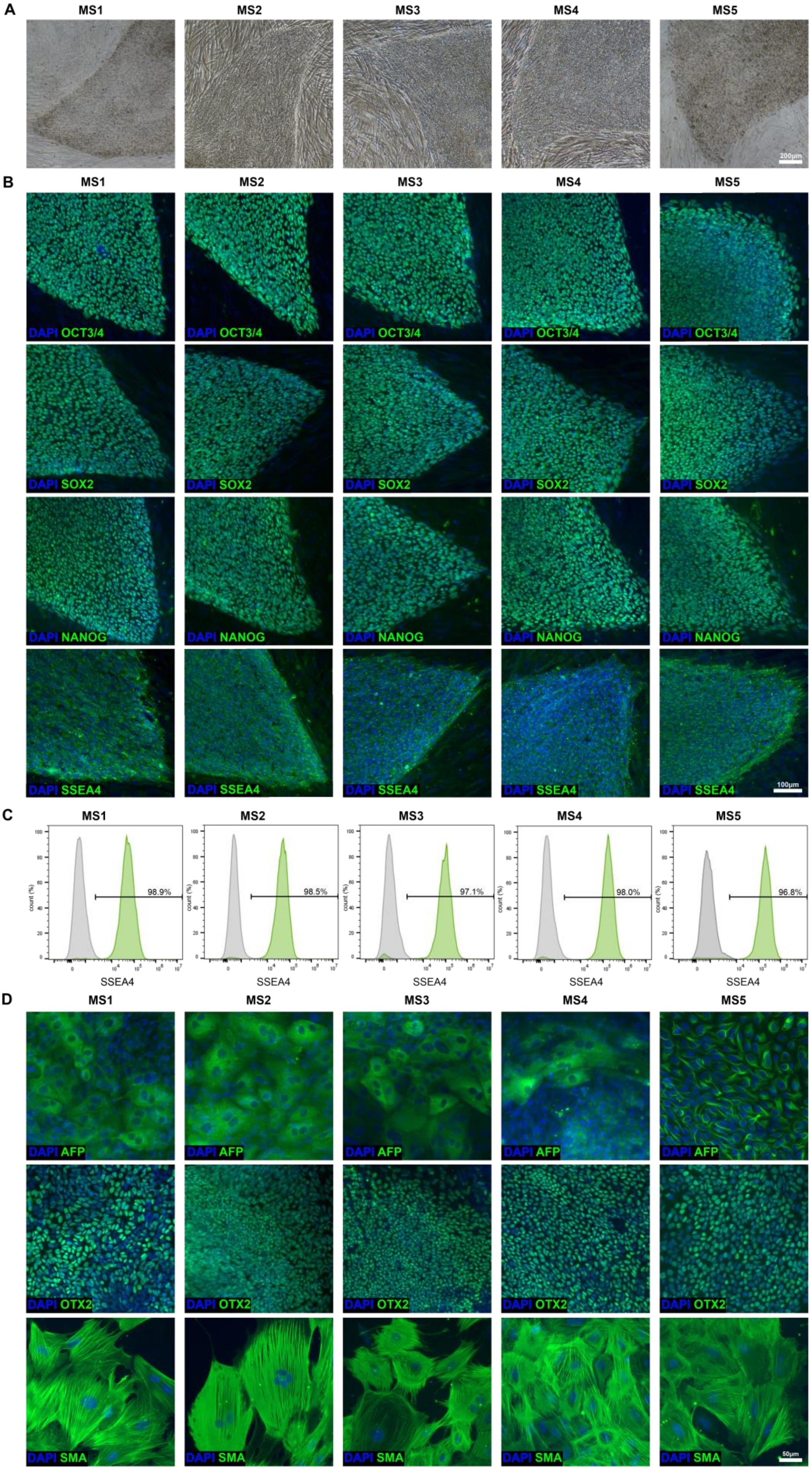
A
MS1
MS2
MS3
MS4
MS5
200μm
B
DAPI OCT3/4
DAPI SOX2
DAPI NANOG
DAPI SSEA4
100μm
C
98.9%
98.5%
97.1%
98.0%
96.8%
count (%)
SSEA4
D
DAPI AFP
DAPI OTX2
DAPI SMA
50μm

Supplementary Fig. 1 Characterization of the five MS patient-derived iPSC lines. **A** Representative phase contrast images and **B** images of immunofluorescence staining for OCT3/4, SOX2, NANOG and SSEA4. **C** Flow cytometry analysis of SSEA4 expression. **D** Representative images of AFP, OTX2 and SMA immunofluorescence staining confirmed the capacity of iPSCs to differentiate into the three germ layers *in vitro* through embryoid body formation.

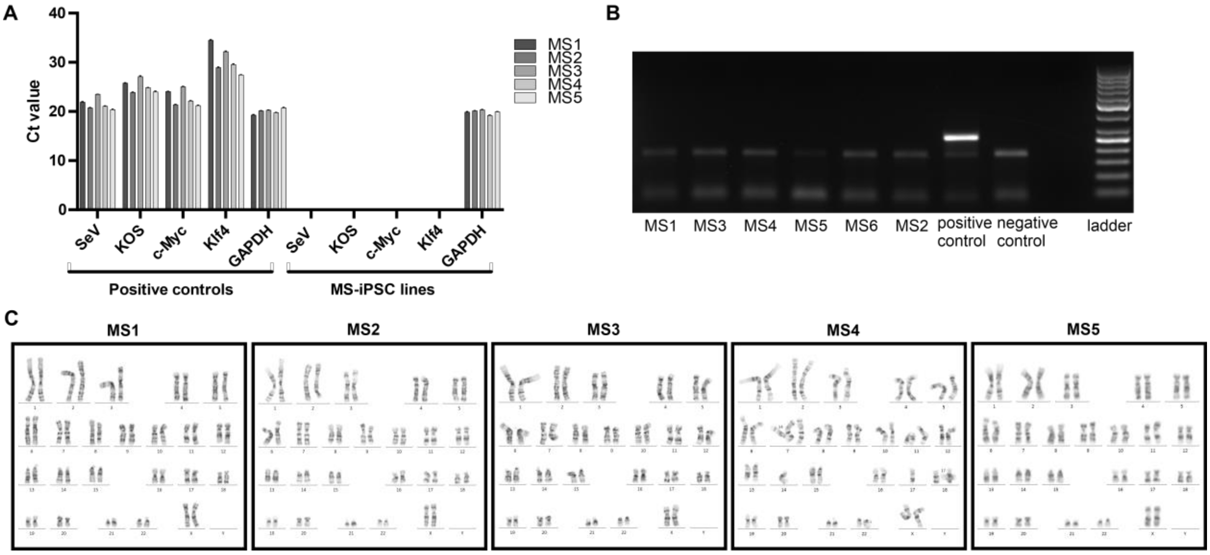

Supplementary Fig. 2 Characterization of MS patient-derived iPSC lines. **A** RT–qPCR analysis of the removal of Sendai virus vectors and transgenes (SeV, KOS, c-Myc and Klf4) from MS iPSCs. Positive controls were collected after transduction (passage 0), and MS-iPSC samples were collected at passage 7–10. **B** Cells were negative for mycoplasma detected via RT–PCR. **C** The karyotype analysis confirmed the normal diploid 46,XX karyotype.

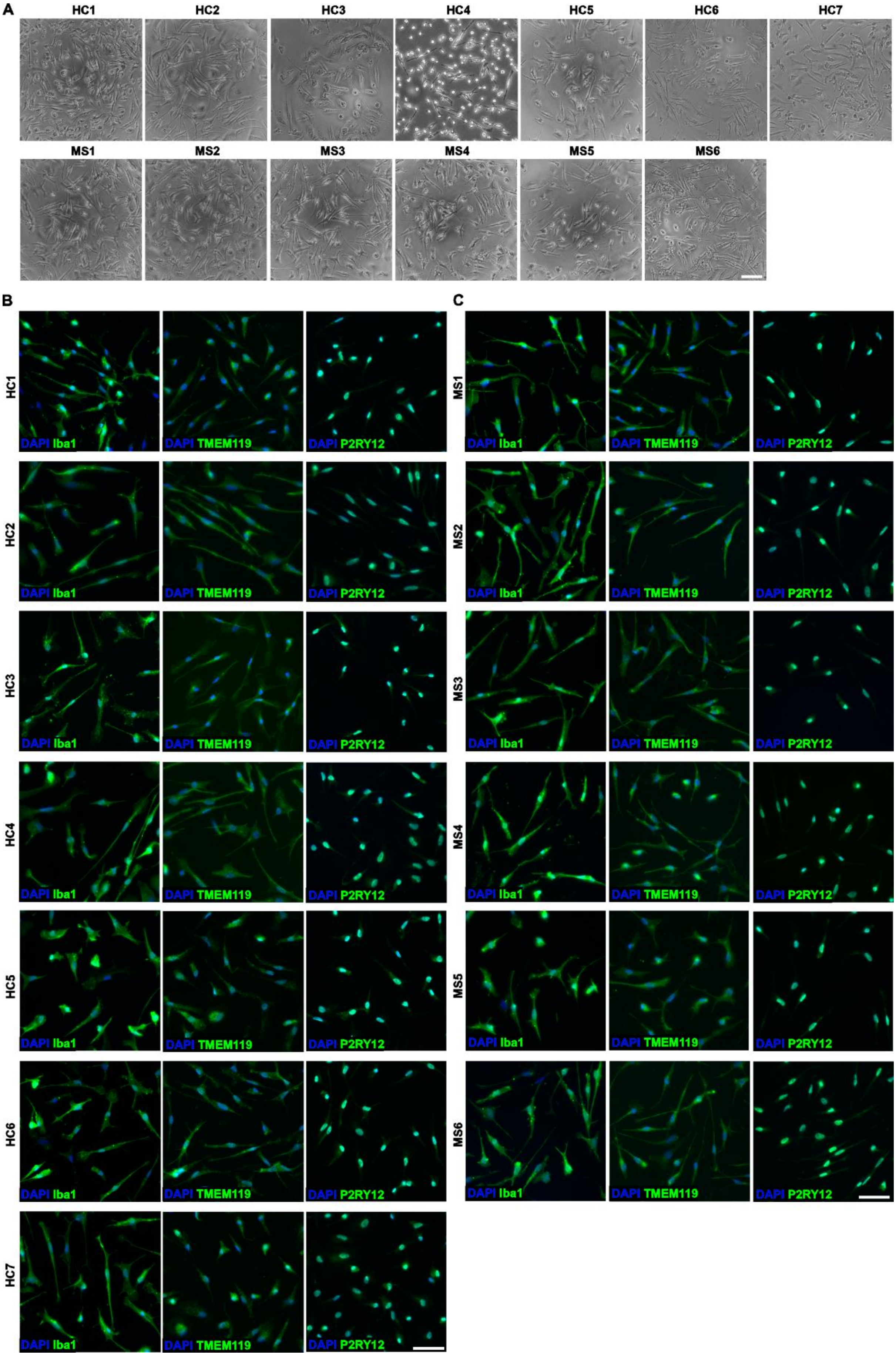

A
HC1
HC2
HC3
HC4
HC5
HC6
HC7
MS1
MS2
MS3
MS4
MS5
MS6
B
C
DAPI Iba1
DAPI TMEM119
DAPI P2RY12

Supplementary Fig. 3 Characterization of HC and MS iPSC-derived iMGLs. **A** Representative phase contrast images of HC and MS iMGLs. Scale bar = 100 µm. Representative images of immunofluorescence staining for Iba1, TMEM119 and P2RY12 in **B** HC and **C** MS iMGLs. Scale bar = 50 µm.

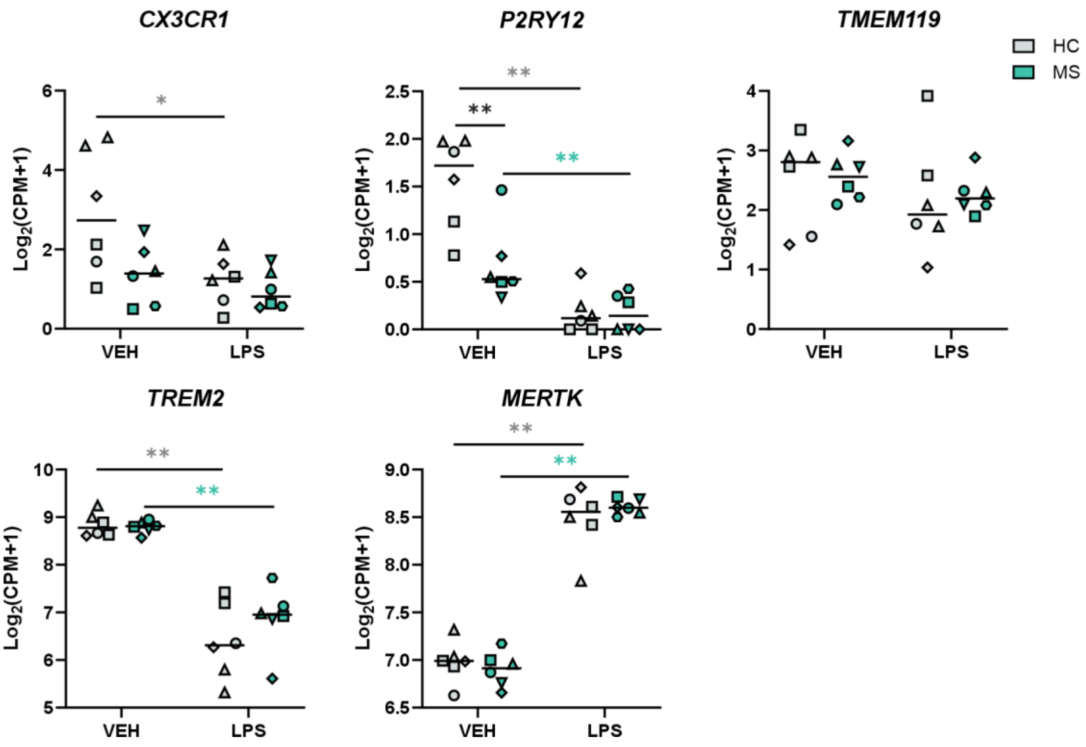

Supplementary Fig. 4 Expression of microglial signature genes *CX3CR1, P2RY12, TMEM119, TREM2* and *MERTK* in RNA-sequencing data of vehicle- and LPS-stimulated HC and MS iMGLs. Data [log2 (CPM+1)] presented as single datapoints and medians. $n = 6$, 4 HC cell lines and 6 MS cell lines, with 1-2 independent differentiations. Different symbols for individual iPSC lines are presented in Supplementary Table 1. Mann–Whitney U test, $*p < 0.05$, and $**p < 0.01$.

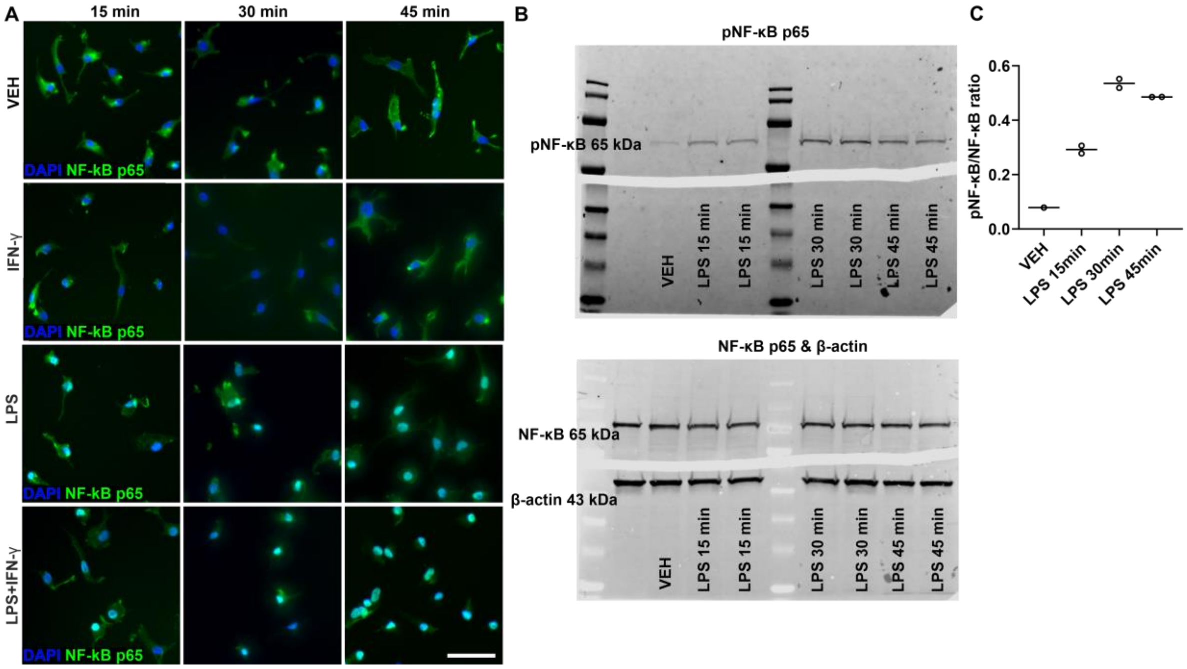

Supplementary Fig. 5 LPS stimulation activates the NF-κB signalling pathway in iMGLs. **A** Representative images of immunofluorescence staining for NF-κB p65 at 15 min, 30 min and 30 min in unstimulated, IFNγ-, LPS- or IFNγ+LPS-stimulated HC and MS iMGLs. Scale bar = 50 µm. **B** Western blots showing the levels of phospho-NF-κB p65, NF-κB p65 and β-actin in unstimulated sample and samples treated with LPS for 15 min, 30 min or 45 min. Nine micrograms of protein was loaded into each lane. **C** Quantification of the phospho-NF-κB p65 and NF-κB p65 expression ratios. Protein levels were normalized to those of β-actin. *n* = 1–2 technical replicates (wells), with 1 differentiation. The data presented as single datapoints and medians.

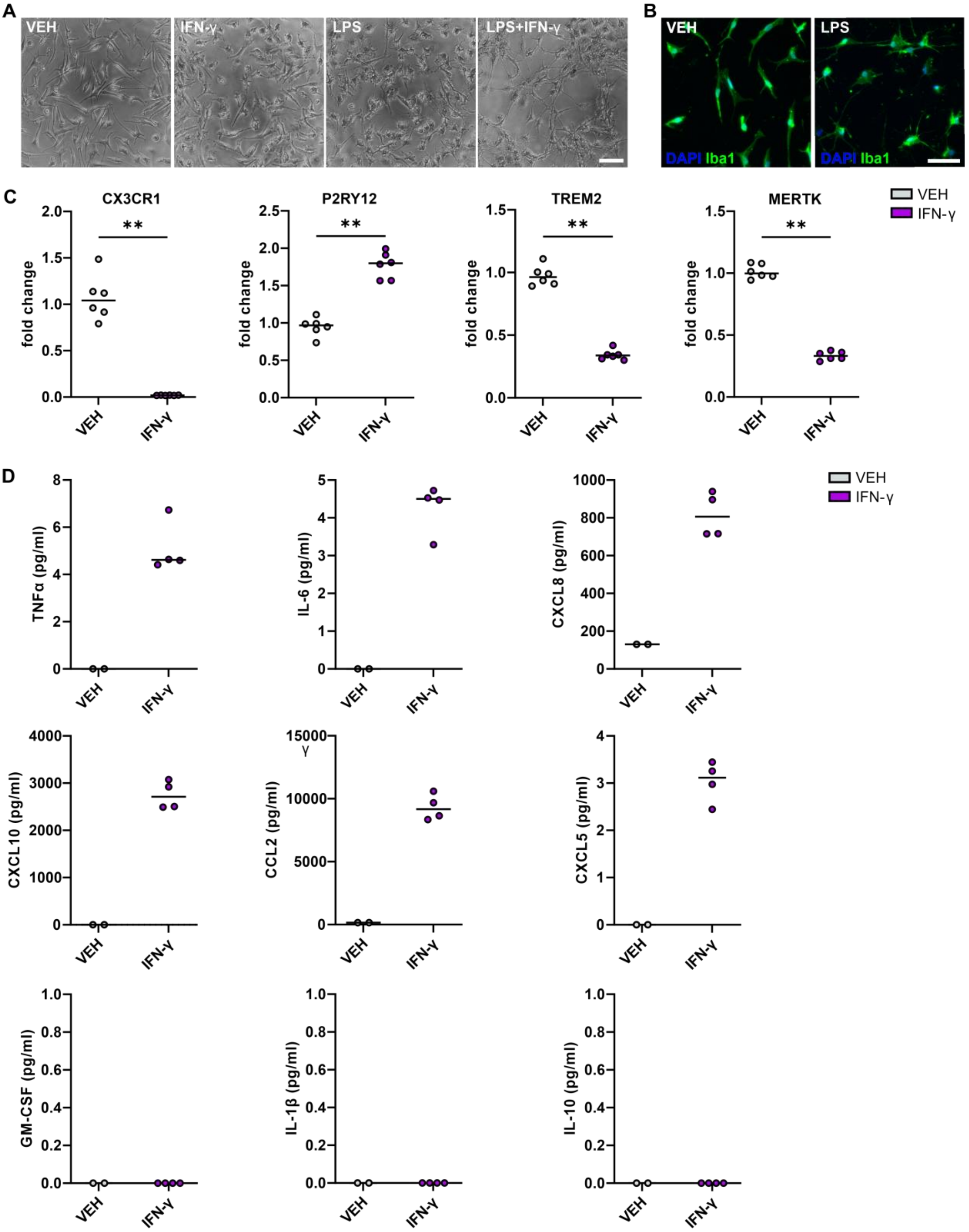

Supplementary Fig. 6 The effect of inflammatory stimulation on the iMGL phenotype. **A** Representative phase contrast images of vehicle- and IFN-γ-, LPS- or IFN-γ+LPS-stimulated iMGLs after 24 h of treatment. Scale bar = 100 µm. **B** Representative images of Iba1 immunofluorescence staining in vehicle- and LPS-stimulated iMGLs after 24 h of treatment. Scale bar = 50 µm. **C** qPCR analysis of the expression of the microglial signature genes

CX3CR1, *P2RY12*, *TREM2* and *MERTK* in vehicle- and IFN-γ-stimulated iMGLs after 24 h of treatment (n = 6 technical replicates, 1 HC cell line, with 1 differentiation). The data are presented as single data points and medians. **D** Analysis of cytokines secreted from vehicle- and IFN-γ-stimulated iMGLs after 24 h of treatment (1 HC cell line, with 1 differentiation). The data are presented as single data points and medians. Mann–Whitney U test, $^{**}p < 0.01$.

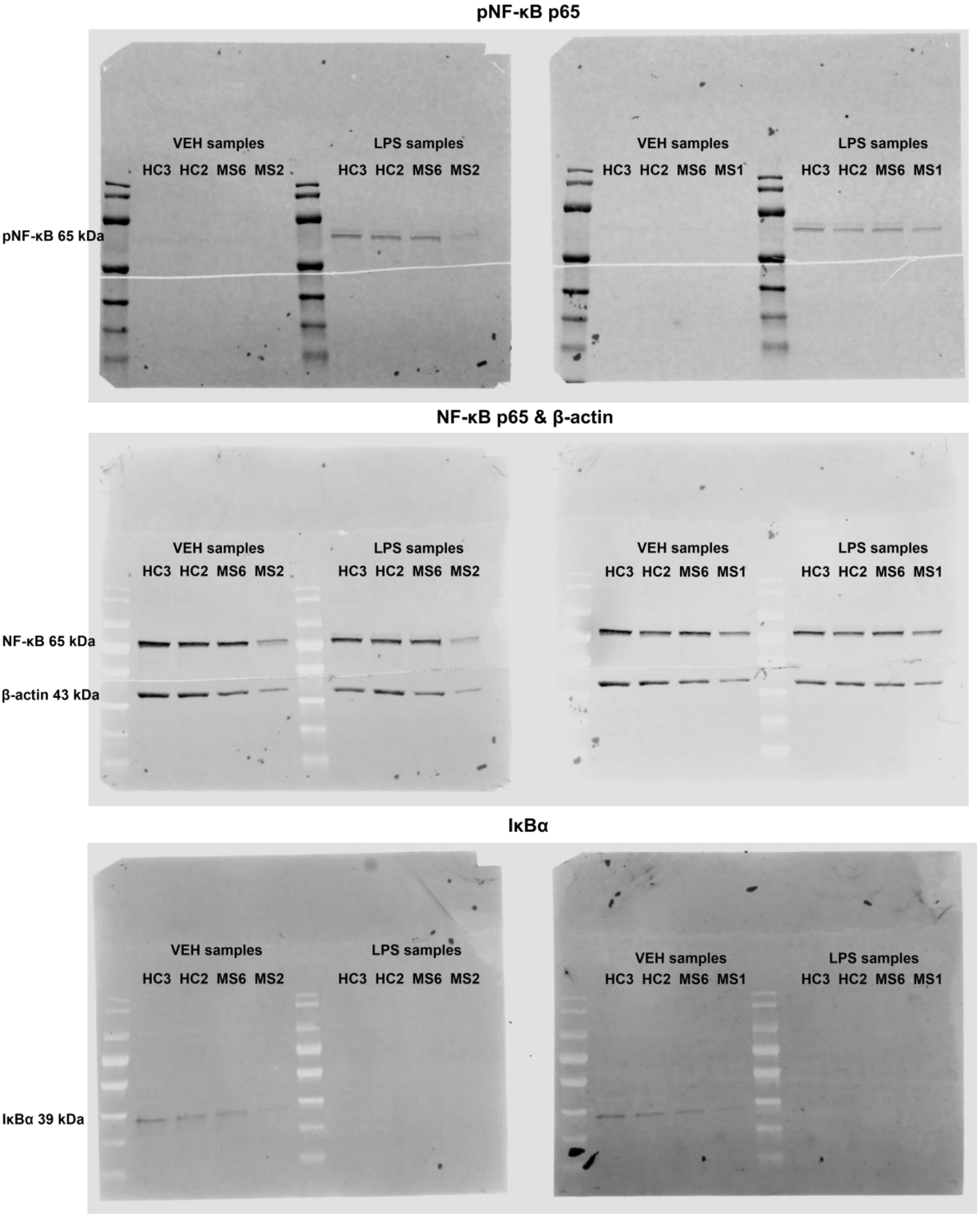

Supplementary Fig. 7 Western blots of NF-κB signalling proteins in HC and MS iMGL samples. Western blots show the levels of phospho-NF-κB p65, NF-κB p65, β-actin and IκBα in vehicle- and LPS-treated (45 min, 20 ng/ml) HC (2 cell lines) and MS iMGLs (3 cell lines); 1-2 technical replicates (wells), 1-2 independent differentiations. Nine micrograms of protein was loaded per lane. See also Figure 3D.

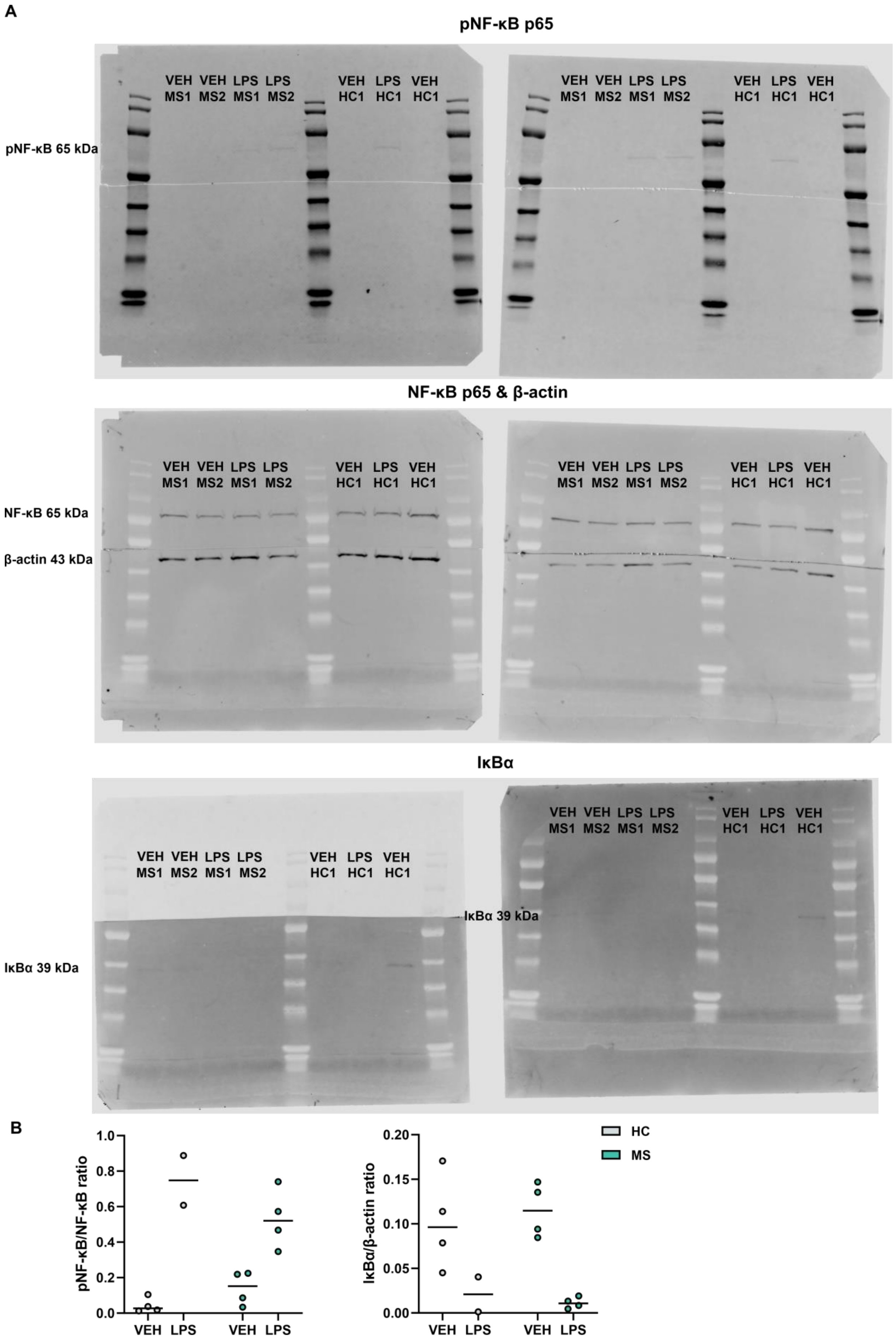
A
pNF-κB p65
VEH MS1
VEH MS2
LPS MS1
LPS MS2
VEH HC1
LPS HC1
VEH HC1
pNF-κB 65 kDa
NF-κB p65 & β-actin
NF-κB 65 kDa
β-actin 43 kDa
IκBα
IκBα 39 kDa
B
pNF-κB/NF-κB ratio
IκBα/β-actin ratio
VEH
LPS
HC
MS

Supplementary Fig. 8 Western blots of NF-κB signalling proteins in HC and MS iMGL samples. **A** Western blots show the levels of phospho-NF-κB p65, NF-κB p65, β-actin and IκBα in vehicle- and LPS-treated (45 min, 20 ng/ml) HC (1 cell line, with 2 independent differentiations) and MS iMGLs (2 cell lines, with 1 differentiation). Five micrograms of protein was loaded per lane. **B** Quantification of the phospho-NFκB p65 and NFκB p65 expression ratios and IκBα protein levels. Protein levels were normalized to those of β-actin. *n* = 2–4 samples, with 2 independent differentiations. The data are presented as single datapoints and medians.

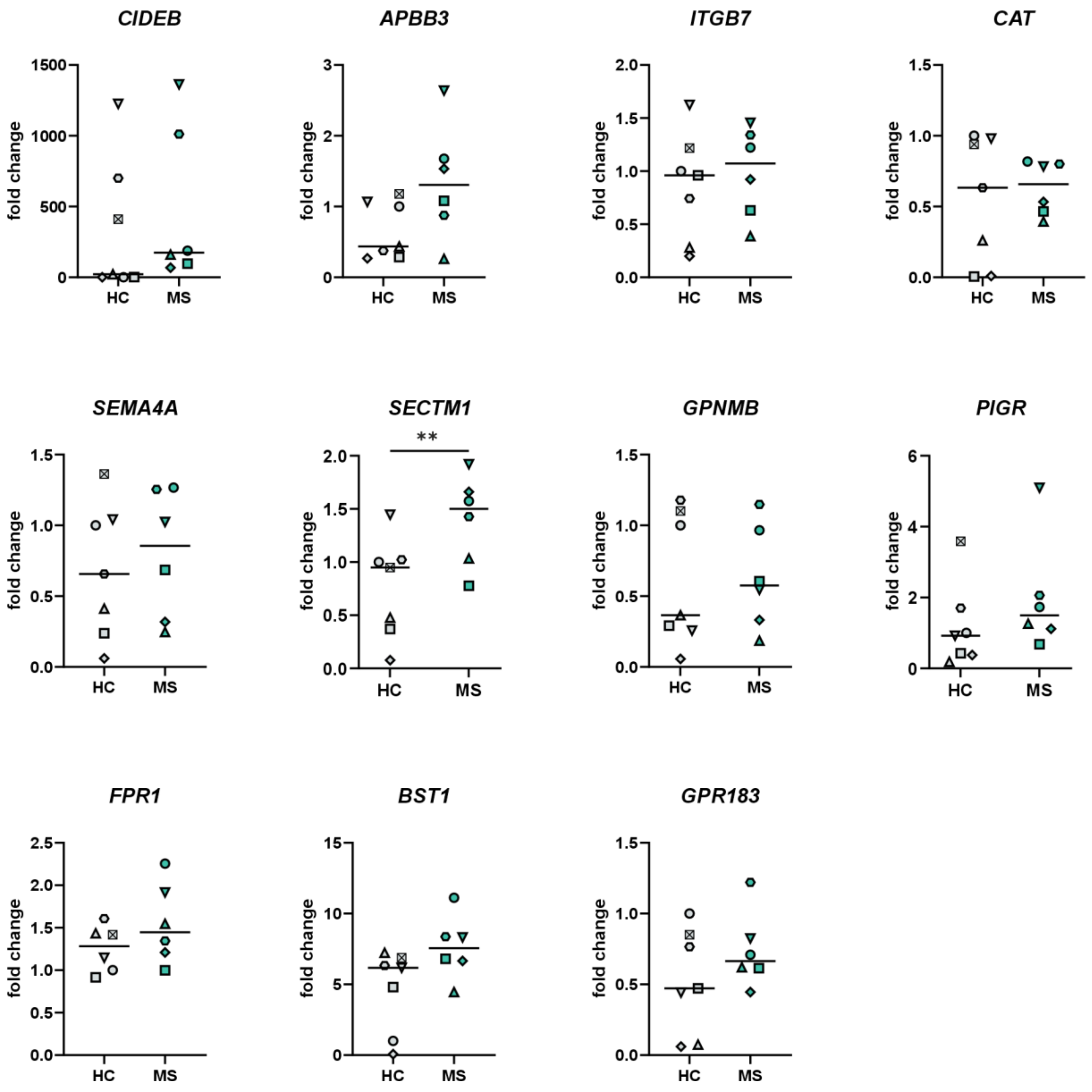

Supplementary Fig. 9 Validation of RNA-seq results by qPCR analysis of selected genes in MS iMGLs compared to HC iMGLs. Fold change values for MS iMGL upregulated genes *CIDEB*, *APBB3*, *ITGB7*, *CAT*, *SEMA4A*, *SECTM1*, *GPNMB*, *PIGR*, *FPR1*, *BST1* and *GPR183*

in HC and MS iMGLs. *n* = 6–7, 7 HC cell lines and 6 MS cell lines. The data are presented as mean values for individual iPSC lines normalized to the HC1. Symbol coding for iPSC lines is shown in Supplementary Table 1. Mann–Whitney U test, *p < 0.05, and **p < 0.01.

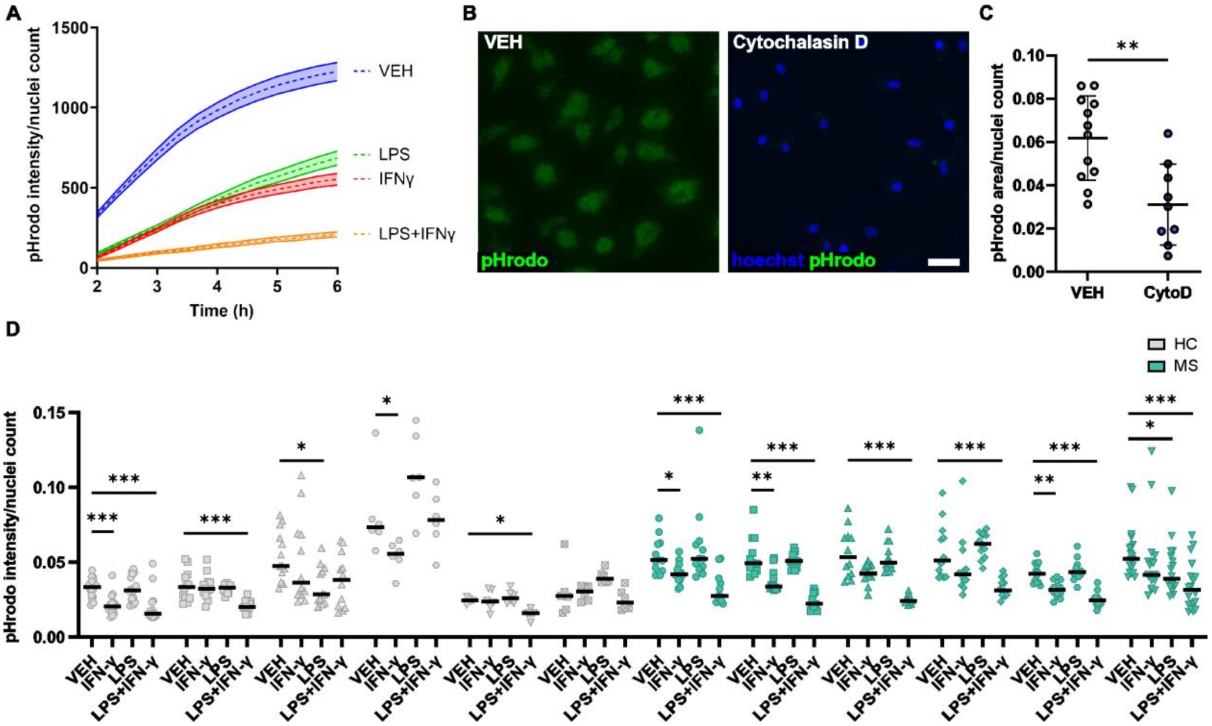

Supplementary Fig. 10 Analysis of iMGL phagocytosis. **A** Representative time curve for the green fluorescence intensity of pHrodo zymosan phagocytosis in vehicle-, IFN-γ-, LPS- or IFN-γ+LPS-stimulated iMGLs after 24 h of treatment. *n* = 18 images per condition and time point; 6 wells/group, 1 differentiation. The data are presented as the means ± SEMs. **B** Representative fluorescence images of pHrodo zymosan bioparticles phagocytosed by iMGLs treated with the vehicle or the phagocytosis inhibitor cytochalasin D for 2 h. Scale bar = 50 µm. **C** Quantification of pHrodo zymosan phagocytosis in vehicle- and cytochalasin D-treated iMGLs. *n* = 9–12 images, 2–6 wells/group, with 2 independent differentiations. The data are presented as single data points and means ± SD. Unpaired t test, ***p* < 0.01. **D** Quantification of pHrodo fluorescence intensity normalized to the number of nuclei for each 6 HC (grey colour) and 6 MS iMGL (green colour) cell line. n = 5–19 images per condition, with 1–3 differentiations per cell line. The data are presented as single data points and medians. The symbol coding of HC and MS iMGL cell lines is presented in Supplementary Table 1. Asterisks indicate statistically significant differences compared with the vehicle treatment within cell

lines, as determined using the Mann–Whitney U test with Bonferroni's correction $*p < 0.05$, $**p < 0.01$, and $***p < 0.001$.

Supplementary Tables

Supplementary Table 1. Human induced pluripotent stem cell lines used in this study.

Symbol*	Cell line	Alias	Sex	Karyo -type	Health status	Origin	Reference
●	MS1	MS1	F	46,XX	MS	PBMC	this article
■	MS2	MS2	F	46,XX	MS	PBMC	this article
▲	MS3	MS3	F	46,XX	MS	PBMC	this article
◆	MS4	MS4	F	46,XX	MS	PBMC	this article
⬣	MS5	MS5	F	46,XX	MS	PBMC	this article
▼	MS6	MS6	F	46,XX	MS	PBMC	Lotila et al. 2022 [1]
○	UTA.04511.WT	HC1	M	46,XY	Healthy	Fibroblast	Ojala et al. 2016 [2]
□	UTA.10902.EURCCs	HC2	F	46,XX	Healthy	Fibroblast	Hongisto et al. 2017 [3]
△	UTA.04602.WT	HC3	F	46,XX	Healthy	Fibroblast	Ojala et al. 2016 [2]
◇	UTA.11311.EURCCs	HC4	F	46,XX	Healthy	Fibroblast	Häkli et al. 2022 [4]
⬡	Hel54.1	HC5	F	46,XX	Healthy	PBMC	Trokovic et al. 2014 [5]
▽	Hel55.5	HC6	F	46,XX	Healthy	PBMC	Trokovic et al. 2014 [5]
⊠	Hel96.6	HC7	F	46,XX	Healthy	PBMC	Trokovic et al. 2014 [5]

Abbreviations: HC, healthy control; MS, multiple sclerosis; F female; M male; PBMC peripheral mononuclear cells.
*Symbols indicate the iPSC lines used in the figures.

Supplementary Table 2. qPCR primers used to characterize iMGLs.

Target	Cat no.	Supplier
APBB3	Hs00195923_m1	Thermo Fisher Scientific
BST1	Hs01070189_m1	Thermo Fisher Scientific
CAT	Hs00156308_m1	Thermo Fisher Scientific
CD74	Hs00269961_m1	Thermo Fisher Scientific
CIDEB	Hs00205339_m1	Thermo Fisher Scientific
CX3CR1	Hs04187059_m1	Thermo Fisher Scientific
FPR1	Hs00181830_m1	Thermo Fisher Scientific
GPNMB	Hs01095669_m1	Thermo Fisher Scientific
GPR183	Hs00953886_m1	Thermo Fisher Scientific
HLA-DPA1	Hs00410276_m1	Thermo Fisher Scientific
HLA-DRA	Hs00219575_m1	Thermo Fisher Scientific
HLA-DRB1	Hs04192464_mH	Thermo Fisher Scientific
ITGB7	Hs01565750_m1	Thermo Fisher Scientific
MERTK	Hs01031979_m1	Thermo Fisher Scientific
P2RY12	Hs00224470_m1	Thermo Fisher Scientific
PIGR	Hs00922561_m1	Thermo Fisher Scientific
SECTM1	Hs00356334_m1	Thermo Fisher Scientific
SEMA4A	Hs00223617_m1	Thermo Fisher Scientific
TMEM119	Hs01938722_u1	Thermo Fisher Scientific
TREM2	Hs00219132_m1	Thermo Fisher Scientific

References

[1] Lotila J, Hyvärinen T, Skottman H, Airas L, Narkilahti S, Hagman S. Establishment of a human induced pluripotent stem cell line (TAUi008-A) derived from a multiple sclerosis patient. Stem Cell Research 2022;63:102865. https://doi.org/10.1016/j.scr.2022.102865.

[2] Ojala M, Prajapati C, Pölönen R-P, Rajala K, Pekkanen-Mattila M, Rasku J, et al. Mutation-Specific Phenotypes in hiPSC-Derived Cardiomyocytes Carrying Either Myosin-Binding Protein C Or α-Tropomyosin Mutation for Hypertrophic Cardiomyopathy. Stem Cells International 2016;2016:1684792.

[3] Hongisto H, Ilmarinen T, Vattulainen M, Mikhailova A, Skottman H. Xeno- and feeder-free differentiation of human pluripotent stem cells to two distinct ocular epithelial cell types using simple modifications of one method. Stem Cell Res Ther 2017;8:291. https://doi.org/10.1186/s13287-017-0738-4.

[4] Häkli M, Kreutzer J, Mäki A-J, Välimäki H, Cherian RM, Kallio P, et al. Electrophysiological Changes of Human-Induced Pluripotent Stem Cell-Derived Cardiomyocytes during Acute Hypoxia and Reoxygenation. Stem Cells Int 2022;2022:9438281. https://doi.org/10.1155/2022/9438281.

[5] Trokovic R, Weltner J, Nishimura K, Ohtaka M, Nakanishi M, Salomaa V, et al. Advanced Feeder-Free Generation of Induced Pluripotent Stem Cells Directly From Blood Cells. Stem Cells Transl Med 2014;3:1402–9. https://doi.org/10.5966/sctm.2014-0113.
